# Response of Bone Metabolism Markers to Ice Swimming in Regular Practitioners

**DOI:** 10.3389/fphys.2021.731523

**Published:** 2021-11-26

**Authors:** Shuai Mu, Yang Xia, Qijun Wu, Chao Ji, Huixu Dai, Ming Zhang, Jiao Jiao, Feng Shi, Shengye Liu, Guangbin Wang, Tao Shen, Ye Tian, Liqing Yang, Qin Fu, Yuhong Zhao

**Affiliations:** ^1^Department of Orthopedics, Shengjing Hospital of China Medical University, Shenyang, China; ^2^Department of Clinical Epidemiology, Shengjing Hospital of China Medical University, Shenyang, China; ^3^Department of Rehabilitation, Shengjing Hospital of China Medical University, Shenyang, China; ^4^Center of Reproductive Medicine, Shengjing Hospital of China Medical University, Shenyang, China; ^5^Department of Health Management, Shengjing Hospital of China Medical University, Shenyang, China

**Keywords:** bone (re)modeling markers, parathyroid hormone (PTH), bone mineral density, exercise, cold exposure

## Abstract

**Objective:** Both exercise and cold exposure cause physiological stress and they often occur in combination. However, the effects of exercise during severe cold on variation in bone metabolism in humans have remained elusive. The aim of this study was to investigate the variations in circulating bone metabolism markers after ice swimming (IS).

**Methods:** Eighty-seven women and men aged 42–84 years old were recruited to perform regular IS activities. Serum parathyroid hormone (PTH), total calcium (Ca^2+^), total phosphorus (Pi), total magnesium (Mg^2+^), N-terminal osteocalcin (N-MID), total propeptide of procollagen 1 (TPINP), and C-terminal telopeptide of type 1 collagen (β-CTX) were measured 30 min before and 30 min after IS. Bone mineral content (BMC) and bone mineral density (BMD) were assessed at lumbar spine 1–4 (L1–L4) and femoral neck (FN). The IS habits were obtained from questionnaires and the 10-year probability of osteoporotic fracture was calculated using the FRAX^®^ tool with and without a BMD value of the FN.

**Results:** There were significant increases in PTH (median, 40.120–51.540 pg/mL), Ca^2+^ (median, 2.330–2.400 mmol/L), and Pi (median, 1.100–1.340 mmol/L) and significant decreases in TPINP (median, 38.190–36.610 ng/mL) and β-CTX (median, 0.185–0.171 ng/mL), while there was a trend for increased serum Mg^2+^ (*P* = 0.058) but no significant change in N-MID (*P* = 0.933) after IS in all subjects. The increases in the proportions of cases of hyperparathyroidemia, hypercalcemia, and hyperphosphatemia in those performing IS were statistically significant. The baseline levels and the changes of bone metabolism markers had associations with osteoporosis and bone status, but these may be age and sex dependent. Finally, there were significant correlations among the bone metabolism markers.

**Conclusion:** IS caused significant alterations in bone metabolic markers, specifically, increases in PTH, Ca^2+^ and Pi should raise concerns about potential cardiovascular health risks in severe cold exercise. Additionally, a divergence between PTH elevation and a decline in bone turnover, which shown a special change of bone metabolism after IS and may suggest potential therapeutic implications of cold exercise in PTH and bone metabolic disorders.

## Introduction

Both exercise and cold exposure cause physiological stress and they often occur in combination. Wintertime sporting events, exercise in cold water, and activities in extreme environmental conditions may all involve this combined stress. The physiological responses to exercise in the cold could be either beneficial or detrimental to health (Tipton et al., 2017). Thus, there is a need to understand in more detail the stress responses under such exceptional conditions. At present, established physiological responses to exercise in the cold are mainly confined to the cardiovascular system, pulmonary system, immune system, and metabolic system (LaVoy et al., 2011; Carlsen, 2012; Tipton et al., 2017; Valtonen et al., 2018). The identified changes are associated with variations in catecholamines, inflammatory cytokines, erythrocytes, leucocytes, and immunoglobulins in circulation after adaptation to cold exercise (LaVoy et al., 2011; Tipton et al., 2017; Checinska-Maciejewska et al., 2019; Knechtle et al., 2021). It has been established that both exercise and cold exposure can cause changes in bone and mineral metabolism (Vainionpaa et al., 2009; Robbins et al., 2018; Dolan et al., 2020), but the effects of severe cold exercise on variations in bone metabolism in humans have remained elusive.

Exercise perturbs calcium-phosphorus metabolism (Lombardi et al., 2020). For instance, parathyroid hormone (PTH), the primary hormone maintaining calcium-phosphate homeostasis, was shown to be stimulated by exercise (Lombardi et al., 2020). However, the effect of exercise on PTH concentrations differed depending on the mode, intensity, and duration of exercise (Maïmoun et al., 2005). In general, significant increases in PTH are observed when an exercise stimulation threshold in terms of intensity (e.g., 15% above the ventilatory threshold for more than 50 min) and/or duration (e.g., 50% VO_2_ max for ≥ 5 h) is exceeded, whereas unsufficient exercise stress (e.g., 15% below the ventilatory threshold for 50 min) does not cause a PTH response (Bouassida et al., 2006; Lombardi et al., 2020). Similarly, bone remodeling, a physiological process that couples bone resorption by osteoclasts to bone formation by osteoblasts, and maintains normal mature bone functions and homeostasis throughout life, was also disturbed by different exercise types, intensities, and durations (Dolan et al., 2020). Ordinarily, acute exercise of sufficient intensity and/or duration often activates bone catabolism (increase of C-terminal telopeptide of type 1 collagen, β-CTX), while bone anabolism (N-terminal propeptide of type 1 procollagen, PINP) appears to be largely unresponsive to exercise (Dolan et al., 2020). In contrast, longitudinal adaptation exercise (training) might stimulate chronic upregulation of bone formative processes (PINP increase), whereas bone resorption (β-CTX level) is less responsive to longitudinal exercise or is even reduced in response to it (Dolan et al., 2020). Bone anabolism and catabolism are altered in different exercise types, and mechanical or metabolic stimuli ultimately lead to different bone outcomes (Proia et al., 2021). For instance, resistance and impact exercise, such as high-impact jumping with enough ground reaction forces (GRFs) (Nguyen, 2018; Proia et al., 2021) and weight-bearing sports like football (Vlachopoulos et al., 2017, 2018) are generally accompanied by increased BMD and/or enhanced bone architecture, while lower-impact sports (e.g., swimming, Gomez-Bruton et al., 2016; Vlachopoulos et al., 2017, 2018; cycling, Vlachopoulos et al., 2017, 2018) usually don’t exert the osteogenic action. The mechanism behind changes in bone metabolism caused by exercise is complex, and the influencing factors include age, sex, nutrition, genetics, training status, and the characteristics of the specific exercise stimulus (Dolan et al., 2020). Nevertheless, monitoring of bone (re)modeling markers (e.g., PINP and β-CTX) could boost knowledge of the dynamic bone response to exercise (Bauer et al., 2012; Vasikaran and Chubb, 2016; Dolan et al., 2020).

Despite a broad range of exercise types having been investigated with regard to the response of bone metabolism (Vainionpaa et al., 2009), the ambient temperature at which exercise is performed should also not be overlooked, as altered environmental temperatures impact on the typical signaling mechanisms during exercise (Febbraio, 2001; Nimmo, 2004). Specifically, cold exposure may have synergistic effects when occurring in combination with exercise to cause certain metabolic perturbations, such as adrenergic humoral stimulation and adipose tissue browning (Doubt, 1991; Jett et al., 2006; Peres Valgas da Silva et al., 2019). Additionally, cold exposure can also cause changes in bone metabolism and morphology through increasing sympathetic tone and augmenting brown adipose tissue (BAT) (Robbins et al., 2018). It is generally accepted that activation of the sympathetic nervous system (SNS) by cold stress damages the bone architecture (Kajimura et al., 2011), whereas BAT induced by cold positively affects bone mass (Lee et al., 2013), however, BAT was shown to be insufficient to prevent cold stress-induced bone loss (Robbins et al., 2018). Actually, the majority of studies suggest that cold exposure contributes to bone loss by inhibiting osteogenesis and enhancing osteoclastic metabolism (Patel et al., 2012; Doucette and Rosen, 2014; Robbins et al., 2018). In contrast, some data demonstrate a positive association between cold stress and bone metabolism. For instance, *in vitro* studies showed that hypothermia could upregulated the key osteoblast transcription factor runt-related transcription factor 2 (Runx2) and osterix expression, resulting in the enhancement of osteoblasts bone formation (Aisha et al., 2014; Nie et al., 2015). Similarly, effects of cold exposure on bone repair and bone formation were also observed through *in vivo* studies (Galliera et al., 2013; Castano et al., 2019).

Taking these findings together, both exercise and cold exposure cause bone metabolic responses, but there are currently major gaps in our understanding of the combined effect of them on bone, especially in the dynamic processes in the human body. Ice swimming (IS), which is defined as swimming in water with a temperature of 5°C or less, is gaining popularity among people living at high latitudes and in cold regions (Knechtle et al., 2021). In 2009, the International Ice Swimming Association (IISA) was formed and, in 2014, IS became a Winter Olympics demonstration sport in Sochi, Russia (Knechtle et al., 2021). IS is the most common cold exercise in cold regions. As such, in the present study, IS was chosen to evaluate the response of bone metabolism to severe cold exercise. The aim of this study is to investigate the variations in circulating bone metabolism markers after IS.

## Materials and Methods

### Participants and Study Design

To address the study goals, we conducted an observational study. The subjects were IS enthusiasts, not newcomers to the sport, aged over 40 years who volunteered to participate in the study. The exclusion criteria included: (1) a failure to provide written informed consent; (2) mental illness or an inability to cooperate with the examination; (3) a history of cardiovascular or cerebrovascular diseases, such as myocardial infarction, serious arrhythmia, or stroke; (4) a diagnosis of osteoporosis; (5) the use of anti-osteoporosis drugs, such as bisphosphonates; and (6) any health risks potentially associated with the study. A total of 87 ice swimmers (90 ice swimmers volunteered to participate, however, three participants who failed to complete the study were excluded) enrolled in this study. These 87 individuals were women and men who were recruited from Liaoyang and Shenyang cities, Liaoning Province, in northeast China.

The research process was as previously described (Mu et al., 2020). Briefly, all participants were divided into three groups, and each group performed the exercise session at a different place and on a different day. The three research times were the winter swimming bases of the Liaoyang ice swimmers on January 12, 2019, and in Shenyang on January 26 and February 16, 2019. The length of the three study lanes was different, but they were all 25–30 meters. The water temperature during the three winter swimming exercises was less than 5°C, which defined the swimming as IS (Knechtle et al., 2021). On the day of the IS, no participants were required to fast, and all underwent the following in sequence: had blood drawn (30 min before IS), performed warm-up preparations onshore, performed IS (14:00 h, according to their own typical IS schedule; swimming time and distance were not mandated), went ashore to perform exercises to recover the body temperature, and then had blood drawn again (30 min after IS). The warm-up and recovery exercises followed each participant’s normal habits. These recovery exercises usually included rope skipping, running, and push-ups. It is important to note that IS is a mixed sport (including warm up and recovery temperature resistance exercise and swimming). The day after the IS, questionnaires, anthropometric analyses, and dual-energy X-ray absorptiometry (DXA) were conducted at the Clinical Nutrition Outpatient and Bone Density Testing Laboratory of Shengjing Hospital. We asked the participants to perform IS according to their own IS distance and time habits on the first day, and reported the distance and time on the questionnaire for the second day. The swimming speed of each participant was different when they were in cold water; however, most participants reported that they took 2–3 min to swim a circle (50–60 m), and 75.86% of the participants (*n* = 87) swam one circle. Hence, in this study, we did not accurately investigate the swimming time of each participant in icy water; however, the “distance swum per session” obtained from the questionnaires represented to some extent the participants’ swimming time in cold water. The study protocol was approved by Shengjing Hospital Ethics Committee of China Medical University and written informed consent was obtained from all participants.

### Anthropometrics

The participants were barefoot and wore only underclothes during the height and body weight measurements. Measurements were performed on an ultrasonic electronic height and weight scale (DHM-200; Dingheng Electronic Technology Co., Ltd., Zhengzhou, China). Body mass index (BMI) was calculated as body weight divided by height squared.

### Bone Density Measurements and Osteoporosis Diagnosis

Lumbar spine (L1–L4) and proximal femur DXA scans were performed by the same method of dual-energy X-ray absorptiometry (Discovery-Wi S/N 88155; Hologic, Boston, MA, United States). To reduce the likelihood of error, the same experienced operator performed all scans in accordance with standardized procedures. The coefficient of variation was under 1.0% for BMC and BMD. The T-score was estimated from the lowest measured BMD value. In accordance with the criteria of the World Health Organization (WHO), a T score above –1 was considered normal, a score between –1 and –2.5 was indicative of osteopenia, and a score below –2.5 was diagnosed as osteoporosis (NIH Consumption Development Panel on Osteoporosis Prevention, Diagnosis, and Therapy, 2001).

### Questionnaires and Fracture Probability Calculations

Detailed questionnaires were completed by all of the participants. The data recorded included age, sex, previous fracture history, history of hip fracture in the parents, current smoking status, administration of adrenocortical hormone, history of rheumatoid arthritis, history of secondary osteoporosis, and alcohol consumption. The 10-year probability of a major osteoporotic fracture (composite of the hip, clinical spine, distal forearm, and proximal humerus) and hip fracture was calculated using the China FRAX^®^ tool^1^ with and without a BMD value of the femoral neck.

### Measurements of Serum Bone Metabolism Characteristics

Blood samples were collected by trained nurses between approximately 13:00 h and 15:00 h. Serum samples were obtained by centrifugation and stored at –80°C. Serum PTH was measured by the electrochemiluminescence immunoassay method using a Cobas analyzer (Cobas e602; Roche, Basel, Switzerland). Serum total calcium (Ca^2+^), total phosphorus (Pi), and total magnesium (Mg^2+^) were measured by the methylxylenol blue (MXB) method, ultraviolet absorption of phosphomolybdate method, and xylylazo violet I (XB-I) method, respectively, using an Architect analyzer (Architect ci16200; Abbott, Chicago, IL, United States). The laboratory reference ranges used in this study were as follows: PTH 15–65 pg/ml, Ca^2+^ 2.1–2.55 mmol/L (12–61 years old), Ca^2+^ 2.2–2.5 mmol/L (above 61 years old), and Pi 0.9–1.6 mmol/L. Serum N-terminal osteocalcin (N-MID), total propeptide of procollagen 1 (TP1NP), and β-CTX were measured by the electrochemiluminescence immunoassay method using a Cobas analyzer (Cobas e601; Roche).

### Statistical Analysis

The data were analyzed for normality by the Shapiro–Wilk test. Normally distributed data of continuous variables are presented as mean and standard deviation. Non-normally distributed data of continuous variables are presented as median and first quartile to third quartile. Data of categorical variables are presented as numbers and percentages. The *t*-test, Mann–Whitney *U* test, or chi-squared test was used to evaluate differences in population characteristics between men and women according to the type of variable. The paired samples *t*-test or the Wilcoxon signed-rank test was used to evaluate differences in serum bone metabolism markers between before and after IS according to the type of variable. Moreover, the Wilcoxon signed-rank test was used to evaluate the proportional changes of PTH, Ca^2+^, and Pi responses to IS according to reference ranges. One-way ANOVA, Welch one-way ANOVA, or Kruskal–Wallis one-way ANOVA was used to evaluate differences among the normal bone mass, osteopenia, and osteoporosis groups according to the equality of error variances (Levene’s test) and the type of variable. P for trend was calculated by entering the median value of each category of osteoporosis as a continuous variable. Spearman correlation coefficient analysis was performed to determine the correlations among bone metabolism markers, and between bone metabolism markers and bone mass density status variables. A two-sided *P*-value < 0.05 was considered statistically significant. Statistical analyses were conducted using IBM SPSS Statistics, version 23.0, and EmpowerStats software with the statistical package R version 3.4.3.

## Results

### Population Characteristics and Sex Differences

Table 1 show the characteristics of the 72 male and 15 female ice swimmers participating in this study. There were no differences between the sexes in terms of age, BMI, IS habits, and FRAX^®^ predicted probability of hip fracture risk with and without BMD. None of the female participants smoked or drank alcohol. The women had significantly lower spine and femoral neck BMC and BMD, while they had a significantly higher risk of major osteoporotic fracture than men. Moreover, the women had significantly higher levels of baseline TPINP and the women exhibited a greater decline in ΔTPINP.

**TABLE 1:** Baseline characteristics of the study subjects.

**Characteristic**	**All subjects**	**Male**	**Female**	***P*-value^*a*^**
*N* (%)	87 (100)	72 (82.8)	15 (17.2)	–
Age, *N* (%)				0.088
40–55	32 (36.8)	27 (37.5)	5 (33.3)	
56–65	40 (46.0)	30 (41.7)	10 (66.7)	
≥66	15 (17.2)	15 (20.8)	0 (0.0)	
BMI, median (Q1–Q3), kg/m^2^	25.6 (24.4–27.9)	25.6 (24.7–28.1)	24.3 (23.7–26.9)	0.263
Smokers, *N* (%)	21 (24.1)	21 (29.2)	0 (0.0)	0.016
Drinkers, *N* (%)	45 (51.7)	45 (62.5)	0 (0.0)	<0.001
IS distance per session, *N* (%), meters				0.848
<80 m	74 (85.1)	61 (84.7)	13 (86.7)	
≥ 80 m	13 (14.9)	11 (15.3)	2 (13.3)	
IS frequency per week, N (%)				0.258
≤3 t/w	16 (18.4)	11 (15.3)	5 (33.3)	
4–5 t/w	27 (31.0)	23 (31.9)	4 (26.7)	
6–7 t/w	44 (50.6)	38 (52.8)	6 (40.0)	
Total IS years, *N* (%), years				0.188
<5 years	33 (37.9)	26 (36.1)	7 (46.7)	
5–9 years	25 (28.7)	19 (26.4)	6 (40.0)	
≥10 years	29 (33.3)	27 (37.5)	2 (13.3)	
L1–L4 BMC, mean (SD), g	67.3 (13.2)	70.4 (11.8)	52.8 (9.6)	<0.001
L1–L4 BMD, mean (SD), g/cm^2^	1.0 (0.1)	1.0 (0.1)	0.9 (0.2)	0.002
FN BMC, median (Q1–Q3), g	4.3 (3.6–4.8)	4.4 (3.9–4.9)	3.4 (3.0–3.6)	<0.001
FNBMD, median (Q1–Q3), g/cm^2^	0.8 (0.7–0.9)	0.8 (0.7–0.9)	0.7 (0.6–0.8)	<0.001
FRAX HF1, median (Q1–Q3), %	0.5 (0.2–1.0)	0.5 (0.2–1.0)	0.5 (0.3–0.8)	0.623
FRAX HF2, median (Q1–Q3), %	0.4 (0.2–0.8)	0.4 (0.2–0.8)	0.4 (0.2–1.0)	0.366
FRAX MOF1, median (Q1–Q3), %	2.6 (1.8–3.4)	2.5 (1.8–3.1)	3.2 (2.5–5.5)	0.032
FRAX MOF2, median (Q1–Q3), %	2.5 (1.8–3.9)	2.2 (1.7–3.6)	3.6 (2.6–5.6)	0.007
Pre-IS PTH, median (Q1–Q3), pg/mL	40.1 (33.0–48.5)	39.9 (32.1–47.9)	42.1 (36.0–52.1)	0.366
Pre-IS Ca^2+^, median (Q1–Q3), mmol/L	2.3 (2.3–2.4)	2.3 (2.3–2.4)	2.4 (2.3–2.4)	0.434
Pre-IS Pi, median (Q1–Q3), mmol/L	1.1 (1.0–1.2)	1.1 (1.0–1.2)	1.1 (1.1–1.2)	0.563
Pre-IS Mg^2+^, mean (*SD*), mmol/L	1.0 (0.1)	1.0 (0.1)	1.0 (0.1)	0.908
Pre-IS N-MID, median (Q1–Q3), ng/mL	13.9 (11.7–17.5)	13.6 (11.1–16.8)	16.6 (12.2–19.7)	0.064
Pre-IS TPINP, median (Q1–Q3), ng/mL	38.2 (32.1–50.3)	37.2 (31.2–44.4)	53.8 (45.9–81.3)	<0.001
Pre-IS β-CTX, median (Q1–Q3), ng/mL	0.2 (0.1–0.2)	0.2 (0.1–0.2)	0.3 (0.2–0.4)	0.072
ΔPTH, median (Q1–Q3), pg/mL	11.2 (2.9–20.3)	10.0 (2.4–18.4)	16.3 (9.8–21.5)	0.174
ΔCa^2+^, mean (*SD*), mmol/L	0.1 (0.1)	0.1 (0.1)	0.1 (0.1)	0.944
ΔPi, mean (*SD*), mmol/L	0.2 (0.2)	0.3 (0.2)	0.2 (0.1)	0.065
ΔMg^2+^, mean (*SD*), mmol/L	0.0 (0.0)	0.0 (0.0)	0.0 (0.0)	0.384
ΔN-MID, median (Q1–Q3), ng/mL	0.0 (–0.6 to 0.5)	0.0 (–0.5 to 0.5)	–0.0 (–0.6 to 0.5)	0.933
ΔTPINP, median (Q1–Q3), ng/mL	–1.9 (–3.8 to –0.1)	–1.5 (–3.2 to 0.0)	–3.6 (–8.8 to –2.5)	0.005
Δβ-CTX, median (Q1–Q3), ng/mL	–0.0 (–0.0 to 0.0)	–0.0 (–0.0 to 0.0)	–0.0 (–0.0 to 0.0)	0.445

*SD, standard deviation; Q1, first quartile; Q3, third quartile; BMI, body mass index; IS, ice swimming; L1–L4, lumbar spine1–4; FN, femoral neck; BMC, bone mineral content; BMD, bone mineral density; FRAX HF1, FRAX predicted probability of hip fracture (without BMD); FRAX HF2, FRAX predicted probability of hip fracture (with BMD); FRAX MOF1, FRAX predicted probability of major osteoporotic fracture (without BMD); FRAX MOF2, FRAX predicted probability of major osteoporotic fracture (with BMD); Pre-IS, the circulating levels before ice swimming; PTH, parathyroid hormone; N-MID, N-terminal osteocalcin; TPINP, total propeptide of procollagen 1; β-CTX, C-terminal crosslaps; Δ, the circulating changes responses to ice swimming. ^*a*^The *P*-values were obtained using the *t*-test or the Mann–Whitney *U* test or the χ^2^ test to evaluate differences between men and women according to the type of variable.*

### Bone Metabolism Markers in Response to Ice Swimming

In all subjects, IS-induced changes in serum bone metabolism markers are reported in Table 2. After the IS, there were significant increases in PTH (from median, 40.120; Q1–Q3, 32.995–48.475; to median, 51.540; Q1–Q3, 40.925–66.405, pg/mL), Ca^2+^ (from median, 2.330; Q1–Q3, 2.285–2.380; to median, 2.400; Q1–Q3, 2.330–2.465, mmol/L), and Pi (from median, 1.100; Q1–Q3, 1.000–1.185; to median, 1.340; Q1–Q3, 1.200–1.470, mmol/L); and significant decreases in TPINP (from median, 38.190; Q1–Q3, 32.110–50.275; to median, 36.610; Q1–Q3, 29.485–50.260, ng/mL) and β-CTX (from median, 0.185; Q1–Q3, 0.148–0.244; to median, 0.171; Q1–Q3, 0.138–0.249, ng/mL). There was a tendency for increased in serum Mg^2+^ (from mean, 1.007; SD, 0.060; to mean, 1.016; SD, 0.068, mmol/L; *P* = 0.058), but no significant change was found in N-MID (*P* = 0.933) after IS in all subjects. In ≥66 years participants, the increases in PTH after IS were vanished (*P* = 0.156, Supplementary Table 1). Supplementary Table 2 shows there were significant increases in serum Mg^2+^ (*P* = 0.045) in male and no significant change was found in β-CTX (*P* = 0.842) after IS in female.

**TABLE 2:** Variations of bone metabolism characteristics responses to ice swimming.

**Serum characteristic**	***N***	**Pre-IS**	**Post-IS**	***P*-value^*a*^**	**Change (**Δ**)**
PTH, median (Q1–Q3), pg/mL	87	40.120 (32.995–48.475)	51.540 (40.925–66.405)	<0.001	11.190 (2.930–20.260)
Ca^2+^, median (Q1–Q3), mmol/L	87	2.330 (2.285–2.380)	2.400 (2.330–2.465)	<0.001	0.060 (0.010–0.110)
Pi, median (Q1–Q3), mmol/L	87	1.100 (1.000–1.185)	1.340 (1.200–1.470)	<0.001	0.240 (0.130–0.350)
Mg^2+^, mean (*SD*), mmol/L	87	1.007 (0.060)	1.016 (0.068)	0.058	0.009 (0.042)
N-MID, median (Q1–Q3), ng/mL	87	13.940 (11.705–17.525)	13.560 (11.470–17.415)	0.933	0.020 (–0.555 to 0.475)
TPINP, median (Q1–Q3), ng/mL	87	38.190 (32.110–50.275)	36.610 (29.485–50.260)	<0.001	–1.920 (–3.785 to –0.100)
β-CTX, median (Q1–Q3), ng/mL	87	0.185 (0.148–0.244)	0.171 (0.138–0.249)	0.001	–0.012 (–0.031 to 0.003)

*SD, standard deviation; Q1, first quartile; Q3, third quartile; IS, ice swimming; PTH, parathyroid hormone; N-MID, N-terminal osteocalcin; TPINP, total propeptide of procollagen 1; β-CTX, C-terminal crosslaps. ^*a*^The *P*-values were obtained using the paired samples *t*-test or the Wilcoxon signed-rank tests to evaluate differences in serum bone metabolism markers before and after IS according to the type of variable.*

### Changes of the Proportional Responses of Parathyroid Hormone, Ca^2+^, and Pi to Ice Swimming

Table 3 shows the percentage variations of PTH, Ca^2+^, and Pi in different reference intervals between before and after IS among all subjects. Six (6.9%) participants had a PTH outside the reference range before IS, while after IS 26 (29.9%) did so. There was a significant increase in the rate of hyperparathyroidemia after IS (*P* < 0.001). One (1.1%) participant had hypocalcemia and six (6.9%) had hypercalcemia before IS, while no subjects had hypocalcemia and eleven (12.6%) had hypercalcemia after IS, constituting a statistically significant improvement (*P* = 0.034). Before IS, the numbers of those with hypophosphatemia, normal blood phosphorus, and hyperphosphatemia were 9 (10.3%), 77 (88.5%), and 1 (1.1%), respectively. Meanwhile, after IS, no subjects had hypophosphatemia and the number of cases with hyperphosphatemia increased to 5 (5.7%). The improvement of blood phosphorus due to IS was statistically significant (*P* < 0.001).

**TABLE 3:** The proportional changes of PTH, Ca2+, and Pi responses to IS.

**Characteristic**	**Below reference value**		**Within reference value**		**Above reference value**		***P*-value^*a*^**
	**Pre-IS**	**Post-IS**	**Pre-IS**	**Post-IS**	**Pre-IS**	**Post-IS**	
PTH, *n* (%)	0(0%)	0 (0%)	81 (93.1%)	61 (70.1%)	6 (6.9%)	26(29.9%)	<0.001
Ca^2+^, *n* (%)	1(1.1%)	0 (0%)	80 (92.0%)	76 (87.4%)	6 (6.9%)	11(12.6%)	0.034
Pi, *n* (%)	9(10.3%)	0 (0%)	77 (88.5%)	82 (94.3%)	1 (1.1%)	5(5.7%)	<0.001

*IS, ice swimming; PTH, parathyroid hormone. ^*a*^The *P*-values were obtained using the Wilcoxon signed-rank tests to evaluate the proportional changes of PTH, Ca^2+^, and Pi responses to IS according to reference ranges.*

### Biomarkers and Osteoporosis

The baseline levels (pre-IS) and changes of serum bone metabolism markers were grouped according to the osteoporosis criteria, as shown in Table 4, and Supplementary Tables 3–7. Among the pre-IS bone metabolism markers, the levels of Ca^2+^, N-MID, TPINP, and β-CTX were higher with increasing severity of osteoporosis (*P* for trend < 0.05, Table 4) in the total population. Among the changes of bone metabolism markers, there were no associations between osteoporosis status and the changes of bone metabolism markers in response to IS in the total and male populations (Table 4 and Supplementary Table 6). However, in females, the change of blood calcium caused by IS increased with increasing severity of osteoporosis (*P* for trend = 0.033, Supplementary Table 7). In addition, the correlations of bone metabolism markers with bone status parameters are presented in Supplementary Tables 8–13. ΔPTH was negatively correlated with FN BMC in age 55–65 years old participants (Supplementary Table 10), whereas ΔPTH was positively correlated with FN BMC in age ≥ 66 years old participants (Supplementary Table 11).

**TABLE 4:** The differences in bone metabolism characteristics among the normal bone mass, osteopenia, and osteoporosis groups.

**Characteristic**	**Normal (*N* = 37)**	**Osteopenia (*N* = 40)**	**Osteoporosis(*N* = 10)**	***P*-value^a^**	***P* for trend**
Pre-IS PTH, median (Q1–Q3), pg/mL	39.61 (34.36–47.82)	42.70 (33.91–50.59)	35.12 (28.66–43.92)	0.614	0.098
Pre-IS Ca^2+^, median (Q1–Q3), mmol/L	2.32 (2.28–2.35)	2.33 (2.29–2.38)	2.38 (2.31–2.49)	0.114	0.011
Pre-IS Pi, mean (*SD*), mmol/L	1.11 (0.12)	1.11 (0.20)	1.06 (0.17)	0.682	0.585
Pre-IS Mg^2+^, mean (*SD*), mmol/L	1.00 (0.06)	1.01 (0.06)	1.01 (0.06)	0.899	0.692
Pre-IS N-MID, median (Q1–Q3), ng/mL	12.55 (10.98–14.70)	14.63 (11.80–17.55)	17.92 (12.76–21.19)	0.063	0.005
Pre-IS TPINP, median (Q1–Q3), ng/mL	34.61 (29.29–43.57)	40.56 (34.69–49.88)	57.39 (39.46–83.56)	0.020	<0.001
Pre-IS β-CTX, median (Q1–Q3), ng/mL	0.18 (0.16–0.23)	0.19 (0.15–0.24)	0.28 (0.15–0.42)	0.619	0.037
ΔPTH, median (Q1–Q3), pg/mL	11.71 (2.36–20.45)	9.13 (3.35–17.74)	17.46 (6.08–25.34)	0.591	0.892
ΔCa^2+^, mean (*SD*), mmol/L	0.06 (0.07)	0.05 (0.07)	0.10 (0.09)	0.216	0.318
ΔPi, mean (*SD*), mmol/L	0.21 (0.15)	0.24 (0.16)	0.31 (0.16)	0.222	0.092
ΔMg^2+^, mean (*SD*), mmol/L	0.01 (0.04)	0.00 (0.04)	0.02 (0.03)	0.267	0.628
ΔN-MID, mean (*SD*), ng/mL	0.00 (0.98)	0.08 (1.06)	0.34 (1.07)	0.663	0.406
ΔTPINP, median (Q1–Q3), ng/mL	–1.92 (–3.19 to –0.85)	–1.33 (–3.33 to 0.30)	–5.49 (–11.78 to 0.24)	0.284	0.132
Δβ-CTX, median (Q1–Q3), ng/mL	–0.01 (–0.02 to 0.00)	–0.02 (–0.04 to 0.00)	–0.01 (–0.02 to 0.04)	0.457	0.984

*SD, standard deviation; Q1, first quartile; Q3, third quartile; Pre-IS, the circulating levels before ice swimming; PTH, parathyroid hormone; N-MID, N-terminal osteocalcin; TPINP, total propeptide of procollagen 1; β-CTX, C-terminal crosslaps; Δ, the circulating changes responses to ice swimming. ^*a*^The *P*-values were obtained using One-way ANOVA, Welch one-way ANOVA, or Kruskal–Wallis one-way ANOVA was used to evaluate differences among the normal bone mass, osteopenia, and osteoporosis groups according to the equality of error variances (Levene’s test) and the type of variable.*

### Correlations of Biomarkers

**FIGURE 1:**
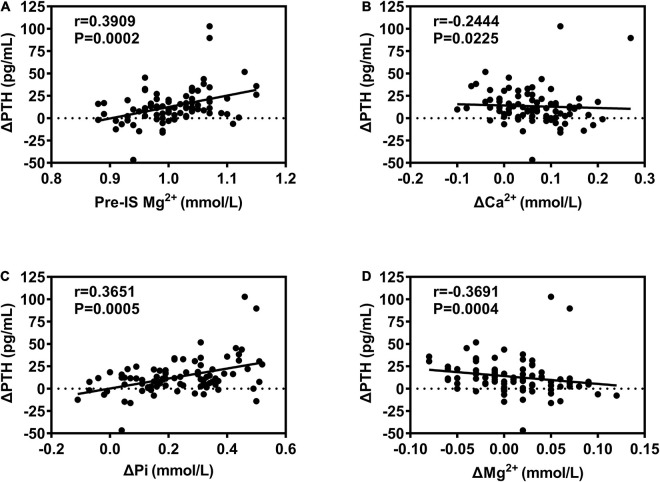
Correlations between ΔPTH and bone metabolism biomarkers. **(A)** Positive correlation between ΔPTH and Pre-IS Mg^2+^; **(B)** negative correlation between ΔPTH and ΔCa^2+^; **(C)** positive correlation between ΔPTH and ΔPi; **(D)** negative correlation between ΔPTH and ΔMg^2+^.

The correlations of biomarkers among all subjects are presented in Figure 1 and Table 5. ΔPTH was positively correlated with pre-IS Mg^2+^ and ΔPi and negatively correlated with ΔCa^2+^ and ΔMg^2+^. ΔCa^2+^ was negatively correlated with ΔPTH and positively correlated with ΔMg^2+^. ΔPi was negatively correlated with pre-IS PTH and Pi and positively correlated with pre-IS Ca^2+^, Mg^2+^, and ΔPTH. ΔMg^2+^ was negatively correlated with ΔPTH and positively correlated with ΔCa^2+^. ΔN-MID was positively correlated with pre-IS Ca^2+^, ΔTPINP, and Δβ-CTX. ΔTPINP was negatively correlated with pre-IS PTH, TPINP, and β-CTX, and positively correlated with ΔN-MID and Δβ-CTX. Δβ-CTX was negatively correlated with pre-IS β-CTX and positively correlated with pre-IS Pi, pre-IS TPINP, ΔN-MID, and ΔTPINP.

**TABLE 5:** Correlations of bone metabolism biomarkers.

**Characteristic**	**Δ PTH**	**Δ Ca^2+^**	**Δ Pi**	**Δ Mg^2+^**	**Δ N-MID**	**Δ TPINP**	**Δβ -CTX**
Pre-IS PTH, (pg/mL)	0.0473	0.0321	−0.2144^*^	–0.0331	–0.0816	−0.2130^*^	–0.0488
Pre-IS Ca^2+^, (mmol/L)	0.1584	–0.1774	0.3022^**^	–0.1075	0.2598^*^	0.1688	0.1507
Pre-IS Pi, (mmol/L)	–0.0722	–0.1062	−0.3824^***^	–0.0437	0.1197	0.0124	0.2151^*^
Pre-IS Mg^2+^, (mmol/L)	0.3909^***^	–0.0023	0.2377*	–0.1176	0.1148	0.1725	–0.0286
Pre-IS N-MID, (ng/mL)	0.0494	–0.1190	0.0117	–0.1429	0.1499	–0.0813	0.1368
Pre-IS TPINP, (ng/mL)	–0.0869	–0.0862	–0.1203	–0.0460	0.1522	−0.2539^*^	0.2298*
Pre-IS β-CTX, (ng/mL)	0.0624	0.0333	–0.0486	0.0304	–0.0639	−0.2508^*^	−0.2503^*^
ΔCa^2+^, (mmol/L)	−0.2444^*^	–	–	–	–	–	–
ΔPi, (mmol/L)	0.3651^***^	0.2102	–	–	–	–	–
ΔMg^2+^, (mmol/L)	−0.3691^***^	0.6022^***^	0.0703	–	–	–	–
ΔN-MID, (ng/mL)	0.1087	–0.0450	–0.0388	0.1340	–	–	–
ΔTPINP, (ng/mL)	0.1771	–0.1436	0.1156	–0.0028	0.5210^***^	–	–
Δβ-CTX, (ng/mL)	0.0879	–0.1674	–0.0587	–0.0629	0.4963^***^	0.2525^*^	–

Pre-IS, the circulating levels before ice swimming; PTH, parathyroid hormone; N-MID, N-terminal osteocalcin; TPINP, total propeptide of procollagen 1; β-CTX, C-terminal crosslaps; Δ, the circulating changes responses to ice swimming; the *P*-values were obtained using Spearman correlation coefficient analysis to determine the correlations among bone metabolism markers. *P < 0.05; **P < 0.01; ***P < 0.001.

## Discussion

In the present study, we chose IS as a representative exercise performed in severe cold to explore the changes of bone metabolism upon cold exercise in humans. We recruited 87 ice swimmers, making this the largest IS study to have been performed. The results showed that IS induced significant increases in circulating PTH and in total calcium and phosphate, there was a tendency for increased serum Mg^2+^ (*P* = 0.058), N-MID remained unchanged, and TPINP and β-CTX decreased significantly. Furthermore, the elevated levels of PTH, Ca^2+^, and Pi exceeded their original reference ranges and the proportions of subjects with hyperparathyroidemia, hypercalcemia and hyperphosphatemia increased after IS. The pre-IS levels of some bone metabolism markers (PTH, Ca^2+^, N-MID, TPINP, and β-CTX) were positively associated with the degree of osteoporosis or fracture risk, and the changes of some bone metabolism markers (Ca^2+^, Pi, and TPINP) also had associations with osteoporosis and bone status, but these may be age and sex dependent. For instance, ΔCa^2+^ was significantly negatively correlated with L1–L4 BMC and BMD, and increased with the severity of osteoporosis (*P* for trend = 0.033) in females. Finally, there were significant correlations among the bone metabolism markers, suggesting potential links between them.

Here, we report that regular IS exercise, involving a warm-up outdoors, 2–3 min of IS, and recovery exercise, could significantly increase PTH levels. The results are consistent with the recent findings of Kovaničová et al. (2020), who also conducted research on IS exercise. In that study, the process of IS was similar to ours, but the subjects spent more time in ice water (10–15 min). This also demonstrated that IS enhanced circulating PTH levels (Kovaničová et al., 2020). Although the evidence is limited, the similar results shown for the different study cohorts indicate that the PTH changes after IS may not be a coincidence and perhaps could be the synergistic effect of exercise and cold stress. Numerous studies have shown that exercise increases PTH secretion (Lombardi et al., 2020), but the existing evidence supports the hypothesis that there is probably a threshold of exercise in terms of intensity and/or duration beyond which PTH is altered; only when the stimulation threshold is exceeded does exercise influence PTH (Maïmoun et al., 2005; Lombardi et al., 2020). We next analyzed the relationship between IS distance per session (represents the intensity and/or duration of IS) and ΔPTH, with no significant relationship being identified (data not shown). Nevertheless, our study has shown that regular IS, identify with whether *via* the high intensity and/or the long duration exercise, could significantly increase PTH levels, suggesting that exercise in a cold environment may enhance the effect of movement on PTH response.

Under physiological conditions, the main mechanism of PTH secretion is modulated by changes in the serum Ca^2+^ concentration, which is detected by the calcium-sensing receptor (CASR) on the major cells of the parathyroid gland (Chavez-Abiega et al., 2020). Thus, it had been considered that the mechanism underlying the exercise-associated rise in PTH is, at least in part, related to the decrease in Ca^2+^ concentration induced by exercise (Bouassida et al., 2006; Lombardi et al., 2020). Indeed, various exercises were shown to be associated with a drop in Ca^2+^ concentration accompanied by an increase in PTH (Thorsen et al., 1997; Townsend et al., 2016), and calcium supplementation before and during exercise partially attenuated the increase of PTH during/after exercise (Barry et al., 2011; Shea et al., 2014). However, PTH has also been observed to exhibit a marked increase despite no change or a rise in calcium concentration during exercise (Scott et al., 2014; Kovaničová et al., 2020; Lombardi et al., 2020). Our results and those from the study by Kovaničová et al. (2020) showed that IS induced increases in circulating PTH along with increases in total calcium and phosphate levels. Meanwhile, Kovaničová et al. (2020) also showed that IS did not cause changes in ionized (albumin-corrected) calcium. Nevertheless, in the study by Kovaničová et al. (2020), it was shown that the increase of PTH was negatively associated with the change in ionized calcium and positively associated with the change in serum total phosphate. Meanwhile, our results demonstrated that ΔPTH was positively correlated with base Mg^2+^ and ΔPi and negatively correlated with ΔCa^2+^ and ΔMg^2+^. The above evidence suggests that calcium disturbance is a factor moderating PTH secretion during/after IS exercise, but not the only one. Actually, phosphate, which is closely related to Ca^2+^, indirectly modulates PTH secretion (Boden and Kaplan, 1990). Mg^2+^ can bind to the same CASR as Ca^2+^, regulating PTH release (Brown and Chen, 1989). Moreover, other influencing factors, such as catecholamine, lactate, PH, leptin, and adiponectin dynamics, all have been discussed in the regulation of PTH response to exercise (Kovaničová et al., 2020). Specifically, cold stress could amplify the responses involving the above factors, for instance, catecholamine, during exercise by activating the SNS (Kvetnansky et al., 2013). Therefore, IS, given the associated cold stimulation, may not require high exercise intensity and/or long exercise duration to stimulate PTH release.

The magnitudes of PTH, calcium, and phosphorus changes may have clinical significance. In this regard, we classified PTH, blood calcium, and phosphorus according to their clinical reference ranges, and found that the proportions of subjects with hyperparathyroidemia and hypercalcemia increased, meanwhile the proportion of subjects with hypophosphatemia decreased and that with hyperphosphatemia increased. Significantly, hyperparathyroidemia, hypercalcemia and hyperphosphatemia all and/or conjoint increased the risk of cardiovascular disease (Larsson et al., 2010; Lutsey et al., 2014; Tournis et al., 2020; Gruson, 2021), which is an important health risk of cold exercise (Manou-Stathopoulou et al., 2015). Consequently, ice swimmers need to be warned of IS induced hyperparathyroidemia, hypercalcemia, and hyperphosphatemia as potential cardiovascular health risks. Although further proof is needed to confirm this, for ice swimmers, who has hyperparathyroidemia, hypercalcemia, and hyperphosphatemia themselves, their blood PTH, Ca^2+^, and Pi levels should be regularly measured and/or they should consider avoid IS. Specifically, our results showed that ΔCa^2+^ increases with the severity of osteoporosis in females, suggesting that female osteoporosis patients may have a higher risk of hypercalcemia after IS. In addition, some drugs can affect serum PTH levels, for instance, the osteoporosis drugs bisphosphonate and denosumab increase the PTH level, while teriparatide decreases its level (Lombardi et al., 2020). Hence, IS plus bisphosphonate or denosumab may intensify hyperparathyroidism to exacerbate the health risk associated with IS.

Bone (re)modeling markers (BMM) are products of bone proteins or cells and represent dynamic bone processes involved in bone anabolism and catabolism (Dolan et al., 2020). BMM can indicate acute bone metabolic responses to exercise, and PINP and β-CTX were recommended as indicators of bone formation and resorption by the National Bone Health Alliance (NBHA) (Bauer et al., 2012; Dolan et al., 2020). Generally, acute vigorous exercise activates osteoclastic metabolism, while bone anabolism appears to be largely unresponsive to this (Dolan et al., 2020). In contrast, the evidence on BMM changes due to cold exposure is limited. To our knowledge, this is the first study to report circulating PINP and β-CTX levels after cold exercise in humans. We found that both PINP and β-CTX levels decreased significantly after IS, suggesting that both anabolism and catabolism (bone turnover) were inhibited after IS, at least in the short term. This is somewhat confusing as, theoretically, PTH is elevated and catecholamines are secreted by SNS activation under cold exercise stress (Kvetnansky et al., 2013), which both activate catabolism (Robbins et al., 2018; Lombardi et al., 2020). However, the PTH axis and SNS are intricately related and little is known about the effect of both signal activations on bone metabolism in humans. Nevertheless, the mechanisms underlying our finding that the suppression of bone turnover after IS may be related to the exercise intensity and/or duration and energy redistribution: (1) BMM response to acute exercise is intensity- and/or duration-dependent (Dolan et al., 2020); despite the addition of cold stress, regular IS exercise in this study was of insufficient intensity and/or duration to activate bone remodeling; and (2) although no dietary restrictions were applied, as IS increased energy expenditure, the body attempted to preserve energy for more critical physiological processes, so bone metabolism may have been restricted. However, these hypotheses need to be confirmed.

Osteocalcin is a non-collagenous protein mainly synthesized by osteoblasts (Hauschka et al., 1989). It participates in the regulation of mineralization and is considered as an osteogenic marker (Brown et al., 1984). In the current study, we did not find a change in N-MID after IS. We speculate that osteogenic mineralization lags behind bone resorption and that the use of a single sampling point may have led to time-specific changes being missed, impacting on the observed findings. On the other hand, it is worth noting that osteocalcin plays important extra-skeletal roles, such as in glucose metabolism, fat metabolism, and muscle activity (Lee and Karsenty, 2008; Lombardi et al., 2015). Thus, besides bone metabolism, changes in osteocalcin may represent metabolic reactions. However, we did not observe responsiveness to osteocalcin during the short recovery window after regular IS exercise.

The basal bone metabolism levels and changes in bone metabolism markers following IS may be related to bone conditions. In this regard, we investigated the relationship between bone metabolism markers and bone status parameters. The results showed that the baseline levels of some bone metabolism markers (PTH, Ca^2+^, N-MID, TPINP, and β-CTX) were positively associated with the severity of osteoporosis or fracture risk, which suggests that osteoporosis is associated with high bone turnover in our cohort. In addition, ΔCa^2+^ was negatively correlated with L1–L4 BMC and BMD, and increased with increasing severity of osteoporosis in females, while ΔPi was negatively correlated with L1–L4 BMC and FN BMC in males, and ΔTPINP was negatively correlated with FRAX^®^ HF2 and FRAX^®^ MOF2 in females. This indicates that different bone status, particularly combined with sex differences, can affect the variations in the levels of some bone metabolism markers caused by IS. Besides, it is worth noting that when we consider age stratification, we found there was no significant change in PTH before and after IS in the age group ≥ 66 years old, and ΔPTH was negatively correlated with FN BMC in age 55–65 years old participants, whereas ΔPTH was positively correlated with FN BMC in age ≥ 66 years old participants. These results suggest that age is also an important impact factor in bone response to IS.

It is well documented that PTH exerts biphasic effects on skeletal homeostasis, with continuous hyperparathyroidism being catabolic, while intermittent treatment boosts bone formation (Wein and Kronenberg, 2018). Evidence has shown that the increase of PTH by strenuous exercise might have an anabolic effect on bone (Maïmoun et al., 2005). In contrast, some data suggested that cold exposure inhibits bone anabolism and leads to bone loss (Patel et al., 2012; Doucette and Rosen, 2014; Robbins et al., 2018). Simultaneously, our results show a divergence between PTH elevation and a decline in bone turnover. Thus, the question arises of what outcomes of IS impact on bone health. We attempted to use multivariate analysis to determine the relationships of IS frequency and years with bone status and metabolism parameters. We found that there were no relationships among IS frequency, total years of IS, and bone status, or relationships to the basal PTH and BMM levels after adjusting for age, sex, and BMI (data not shown). This suggests the skeletal alterations by the IS-induced perturbation of bone metabolic markers are ultimately unclear in the current study, and further research is warranted to answer the above question. On the other hand, PTH may be involved in adipose tissue metabolic and/or thermogenic activation under cold conditions, and Kovaničová et al. (2020) have shown a negative association between the increase of PTH induced by IS and visceral adiposity. We analyzed the relationship between our body composition data (detection method reported previously, Mu et al., 2020) and ΔPTH, and found that ΔPTH is indeed negatively correlated with visceral fat area (*r* = −0.2364, *p* = 0.0275, Spearman’s analysis), and ΔPTH is also negatively correlated with waist to hip ratio (*r* = −0.2285, *p* = 0.0333, Spearman’s analysis). This suggested IS induced PTH changes are beneficial to fat utilization.

This study had several limitations. First, we only investigated bone metabolism markers once after IS, but such single sampling results would fail to capture any dynamic changes of bone metabolism, and would also miss the overall bone remodeling process during the recovery period after IS. Second, we did not correct for plasma volume shifts, which are known to occur during exercise or upon changes in the ambient temperature (Kargotich et al., 1998) and may have impacted our findings. Third, all ice swimmers enrolled in the study were over 40 years, which the specific participants can not represent all age groups’ response of bone metabolism markers to IS. Lastly, no data on pathophysiological/disease state (e.g., menopause, hyperparathyroidism, and cancer), the time interval between lunch and IS, diet on the test day, or dietary habits were included in our study, so we could not rule out effects of metabolic pathologies or feeding/macronutrient status on the outcome.

## Conclusion

The present study conducted in a group of ice swimmers showed significant alterations in bone metabolic markers after IS. In particular, PTH showed marked increases after IS, the underlying mechanisms of which may involve variations in physiological factors (e.g., Ca^2+^, Pi, Mg^2+^, and catecholamines). Increases in PTH, Ca^2+^, and Pi beyond the clinical reference ranges should raise concerns about potential cardiovascular health risks in severe cold exercise. Concurrently, both PINP and β-CTX levels decreased significantly after IS, which suggested that bone turnover was suppressed by IS, which is possibly related to insufficient exercise intensity and/or duration and the redistribution of energy. Finally, although the differences in bone conditions may affect the changes of some bone metabolism markers caused by IS, the impact of long-term IS on the skeletal system is still unclear, which needs to be clarified by more strictly controlled, long-term studies that consider age and sex. Nonetheless, this study expands our understanding of the profile of bone metabolism changes upon cold exercise.

## Data Availability Statement

The raw data supporting the conclusions of this article will be made available by the authors, without undue reservation.

## Ethics Statement

The studies involving human participants were reviewed and approved by Shengjing Hospital Ethics Committee of China Medical University. The patients/participants provided their written informed consent to participate in this study.

## Author Contributions

SM, YX, and YZ designed and supervised the study. SM, MZ, JJ, FS, and SL enrolled the participants, performed the exercise testing, and supervised the training interventions. SM, CJ, YX, HD, and QW analyzed the training and testing data. SM interpreted the results and wrote the manuscript. GW, TS, YT, LY, QF, and YZ reviewed the manuscript and provided important the intellectual content. All authors have read and approved the final manuscript, and agreed with the order of the presentation of the authors.

## Conflict of Interest

The authors declare that the research was conducted in the absence of any commercial or financial relationships that could be construed as a potential conflict of interest.

## Publisher’s Note

All claims expressed in this article are solely those of the authors and do not necessarily represent those of their affiliated organizations, or those of the publisher, the editors and the reviewers. Any product that may be evaluated in this article, or claim that may be made by its manufacturer, is not guaranteed or endorsed by the publisher.

## Footnotes

1
www.sheffield.ac.uk/FRAX®/tool.aspx?country=2

## References

[B1] Aisha M. D. Nor-Ashikin M. N. Sharaniza A. B. Nawawi H. M. Kapitonova M. Y. Froemming G. R. (2014). Short-term moderate hypothermia stimulates alkaline phosphatase activity and osteocalcin expression in osteoblasts by upregulating Runx2 and osterix *in vitro*. *Exp. Cell Res.* 326 46–56. 10.1016/j.yexcr.2014.06.003 24928274

[B2] Barry D. W. Hansen K. C. van Pelt R. E. Witten M. Wolfe P. Kohrt W. M. (2011). Acute calcium ingestion attenuates exercise-induced disruption of calcium homeostasis. *Med. Sci. Sports Exerc.* 43 617–623. 10.1249/MSS.0b013e3181f79fa8 20798655 PMC3145631

[B3] Bauer D. Krege J. Lane N. Leary E. Libanati C. Miller P. (2012). National Bone Health Alliance Bone Turnover Marker Project: current practices and the need for US harmonization, standardization, and common reference ranges. *Osteoporos. Int.* 23 2425–2433. 10.1007/s00198-012-2049-z 22797491 PMC4011662

[B4] Boden S. D. Kaplan F. S. (1990). Calcium homeostasis. *Orthop. Clin. North Am.* 21 31–42. 2404236

[B5] Bouassida A. Latiri I. Bouassida S. Zalleg D. Zaouali M. Feki Y. (2006). Parathyroid hormone and physical exercise: a brief review. *J. Sports Sci. Med.* 5 367–374. 24353453 PMC3842136

[B6] Brown E. M. Chen C. J. (1989). Calcium, magnesium and the control of PTH secretion. *Bone Miner.* 5 249–257. 10.1016/0169-6009(89)90003-2 2655774

[B7] Brown J. P. Delmas P. D. Malaval L. Edouard C. Chapuy M. C. Meunier P. J. (1984). Serum bone Gla-protein: a specific marker for bone formation in postmenopausal osteoporosis. *Lancet* 1 1091–1093. 10.1016/s0140-6736(84)92506-6 6144827

[B8] Carlsen K. H. (2012). Sports in extreme conditions: the impact of exercise in cold temperatures on asthma and bronchial hyper-responsiveness in athletes. *Br. J. Sports Med.* 46 796–799. 10.1136/bjsports-2012-091292 22906782

[B9] Castano D. Comeau-Gauthier M. Ramirez-GarciaLuna J. L. Drager J. Harvey E. Merle G. (2019). Noninvasive Localized Cold Therapy: a New Mode of Bone Repair Enhancement. *Tissue Eng. Part A* 25 554–562. 10.1089/ten.TEA.2018.0191 30187830

[B10] Chavez-Abiega S. Mos I. Centeno P. P. Elajnaf T. Schlattl W. Ward D. T. (2020). Sensing Extracellular Calcium - An Insight into the Structure and Function of the Calcium-Sensing Receptor (CaSR). *Adv. Exp. Med. Biol.* 1131 1031–1063. 10.1007/978-3-030-12457-1_41 31646544

[B11] Checinska-Maciejewska Z. Niepolski L. Checinska A. Korek E. Kolodziejczak B. Kopczynski Z. (2019). Regular cold water swimming during winter time affects resting hematological parameters and serum erythropoietin. *J. Physiol. Pharmacol.* 70 747–756. 10.26402/jpp.2019.5.10 32009627

[B12] Dolan E. Varley I. Ackerman K. E. Pereira R. M. R. Elliott-Sale K. J. Sale C. (2020). The Bone Metabolic Response to Exercise and Nutrition. *Exerc. Sport Sci. Rev.* 48 49–58. 10.1249/JES.0000000000000215 31913188

[B13] Doubt T. J. (1991). Physiology of exercise in the cold. *Sports Med.* 11 367–381. 10.2165/00007256-199111060-00003 1925184

[B14] Doucette C. R. Rosen C. J. (2014). Inducible models of bone loss. *Curr. Protoc. Mouse Biol.* 4 165–180. 10.1002/9780470942390.mo140071 25723184

[B15] Febbraio M. A. (2001). Alterations in energy metabolism during exercise and heat stress. *Sports Med.* 31 47–59. 10.2165/00007256-200131010-00004 11219501

[B16] Galliera E. Dogliotti G. Melegati G. Corsi Romanelli M. M. Cabitza P. Banfi G. (2013). Bone remodelling biomarkers after whole body cryotherapy (WBC) in elite rugby players. *Injury* 44 1117–1121. 10.1016/j.injury.2012.08.057 23000054

[B17] Gomez-Bruton A. Montero-Marín J. González-Agüero A. García-Campayo J. Moreno L. A. Casajús J. A. (2016). The Effect of Swimming During Childhood and Adolescence on Bone Mineral Density: a Systematic Review and Meta-Analysis. *Sports Med.* 46 365–379. 10.1007/s40279-015-0427-3 26607734

[B18] Gruson D. (2021). PTH and cardiovascular risk. *Ann. Endocrinol.* 82 149–150. 10.1016/j.ando.2020.02.005 32192791

[B19] Hauschka P. V. Lian J. B. Cole D. E. Gundberg C. M. (1989). Osteocalcin and matrix Gla protein: vitamin K-dependent proteins in bone. *Physiol. Rev.* 69 990–1047. 10.1152/physrev.1989.69.3.990 2664828

[B20] Jett D. M. Adams K. J. Stamford B. A. (2006). Cold exposure and exercise metabolism. *Sports Med.* 36 643–656. 10.2165/00007256-200636080-00002 16869707

[B21] Kajimura D. Hinoi E. Ferron M. Kode A. Riley K. J. Zhou B. (2011). Genetic determination of the cellular basis of the sympathetic regulation of bone mass accrual. *J. Exp. Med.* 208 841–851. 10.1084/jem.20102608 21444660 PMC3135354

[B22] Kargotich S. Goodman C. Keast D. Morton A. R. (1998). The influence of exercise-induced plasma volume changes on the interpretation of biochemical parameters used for monitoring exercise, training and sport. *Sports Med.* 26 101–117. 10.2165/00007256-199826020-00004 9777683

[B23] Knechtle B. Stjepanovic M. Knechtle C. Rosemann T. Sousa C. V. Nikolaidis P. T. (2021). Physiological Responses to Swimming Repetitive “Ice Miles”. *J. Strength Cond. Res.* 35 487–494. 10.1519/jsc.0000000000002690 29878984

[B24] Kovaničová Z. Kurdiová T. Baláž M. Štefanička P. Varga L. Kulterer O. C. (2020). Cold Exposure Distinctively Modulates Parathyroid and Thyroid Hormones in Cold-Acclimatized and Non-Acclimatized Humans. *Endocrinology* 161:bqaa051. 10.1210/endocr/bqaa051 32242612

[B25] Kvetnansky R. Lu X. Ziegler M. G. (2013). Stress-triggered changes in peripheral catecholaminergic systems. *Adv. Pharmacol.* 68 359–397. 10.1016/b978-0-12-411512-5.00017-8 24054153

[B26] Larsson T. E. Olauson H. Hagström E. Ingelsson E. Arnlöv J. Lind L. (2010). Conjoint effects of serum calcium and phosphate on risk of total, cardiovascular, and noncardiovascular mortality in the community. *Arterioscler. Thromb. Vasc. Biol.* 30 333–339. 10.1161/atvbaha.109.196675 19948843

[B27] LaVoy E. C. McFarlin B. K. Simpson R. J. (2011). Immune responses to exercising in a cold environment. *Wilderness Environ. Med.* 22 343–351. 10.1016/j.wem.2011.08.005 21982757

[B28] Lee N. K. Karsenty G. (2008). Reciprocal regulation of bone and energy metabolism. *Trends Endocrinol. Metab.* 19 161–166. 10.1016/j.tem.2008.02.006 18407515

[B29] Lee P. Brychta R. J. Collins M. T. Linderman J. Smith S. Herscovitch P. (2013). Cold-activated brown adipose tissue is an independent predictor of higher bone mineral density in women. *Osteoporos. Int.* 24 1513–1518. 10.1007/s00198-012-2110-y 22890364 PMC5572572

[B30] Lombardi G. Perego S. Luzi L. Banfi G. (2015). A four-season molecule: osteocalcin. Updates in its physiological roles. *Endocrine* 48 394–404. 10.1007/s12020-014-0401-0 25158976

[B31] Lombardi G. Ziemann E. Banfi G. Corbetta S. (2020). Physical Activity-Dependent Regulation of Parathyroid Hormone and Calcium-Phosphorous Metabolism. *Int. J. Mol. Sci.* 21:5388. 10.3390/ijms21155388 32751307 PMC7432834

[B32] Lutsey P. L. Alonso A. Michos E. D. Loehr L. R. Astor B. C. Coresh J. (2014). Serum magnesium, phosphorus, and calcium are associated with risk of incident heart failure: the Atherosclerosis Risk in Communities (ARIC) Study. *Am. J. Clin. Nutr.* 100 756–764. 10.3945/ajcn.114.085167 25030784 PMC4135486

[B33] Maïmoun L. Simar D. Malatesta D. Caillaud C. Peruchon E. Couret I. (2005). Response of bone metabolism related hormones to a single session of strenuous exercise in active elderly subjects. *Br. J. Sports Med.* 39 497–502. 10.1136/bjsm.2004.013151 16046330 PMC1725278

[B34] Manou-Stathopoulou V. Goodwin C. D. Patterson T. Redwood S. R. Marber M. S. Williams R. P. (2015). The effects of cold and exercise on the cardiovascular system. *Heart* 101 808–820. 10.1136/heartjnl-2014-306276 25673528

[B35] Mu S. Ding D. Ji C. Wu Q. Xia Y. Zhou L. (2020). Relationships Between Circulating Irisin Response to Ice Swimming and Body Composition in People With Regular Exercise Experience. *Front. Physiol.* 11:596896. 10.3389/fphys.2020.596896 33519505 PMC7838676

[B36] Nguyen V. H. (2018). School-based exercise interventions effectively increase bone mineralization in children and adolescents. *Osteoporos. Sarcopenia* 4 39–46. 10.1016/j.afos.2018.05.002 30775541 PMC6362970

[B37] Nie Y. Yan Z. Yan W. Xia Q. Zhang Y. (2015). Cold exposure stimulates lipid metabolism, induces inflammatory response in the adipose tissue of mice and promotes the osteogenic differentiation of BMMSCs *via* the p38 MAPK pathway *in vitro*. *Int. J. Clin. Exp. Pathol.* 8 10875–10886. 26617802 PMC4637617

[B38] NIH Consumption Development Panel on Osteoporosis Prevention, Diagnosis, and Therapy (2001). Osteoporosis prevention, diagnosis, and therapy. *JAMA* 285 785–795. 10.1001/jama.285.6.785 11176917

[B39] Nimmo M. (2004). Exercise in the cold. *J. Sports Sci.* 22 898–915. 10.1080/0264041400005883 15768724

[B40] Patel J. J. Utting J. C. Key M. L. Orriss I. R. Taylor S. E. Whatling P. (2012). Hypothermia inhibits osteoblast differentiation and bone formation but stimulates osteoclastogenesis. *Exp. Cell Res.* 318 2237–2244. 10.1016/j.yexcr.2012.06.021 22771842

[B41] Peres Valgas da Silva C. Hernandez-Saavedra D. White J. D. Stanford K. I. (2019). Cold and Exercise: therapeutic Tools to Activate Brown Adipose Tissue and Combat Obesity. *Biology* 8:9. 10.3390/biology8010009 30759802 PMC6466122

[B42] Proia P. Amato A. Drid P. Korovljev D. Vasto S. Baldassano S. (2021). The Impact of Diet and Physical Activity on Bone Health in Children and Adolescents. *Front. Endocrinol.* 12:704647. 10.3389/fendo.2021.704647 34589054 PMC8473684

[B43] Robbins A. Tom C. Cosman M. N. Moursi C. Shipp L. Spencer T. M. (2018). Low temperature decreases bone mass in mice: implications for humans. *Am. J. Phys. Anthropol.* 167 557–568. 10.1002/ajpa.23684 30187469 PMC7480070

[B44] Scott J. P. Sale C. Greeves J. P. Casey A. Dutton J. Fraser W. D. (2014). Treadmill running reduces parathyroid hormone concentrations during recovery compared with a nonexercising control group. *J. Clin. Endocrinol. Metab.* 99 1774–1782. 10.1210/jc.2013-3027 24476072

[B45] Shea K. L. Barry D. W. Sherk V. D. Hansen K. C. Wolfe P. Kohrt W. M. (2014). Calcium supplementation and parathyroid hormone response to vigorous walking in postmenopausal women. *Med. Sci. Sports Exerc.* 46 2007–2013. 10.1249/MSS.0000000000000320 24576866 PMC4145055

[B46] Thorsen K. Kristoffersson A. Hultdin J. Lorentzon R. (1997). Effects of moderate endurance exercise on calcium, parathyroid hormone, and markers of bone metabolism in young women. *Calcif. Tissue Int.* 60 16–20. 10.1007/s002239900179 9030474

[B47] Tipton M. J. Collier N. Massey H. Corbett J. Harper M. (2017). Cold water immersion: kill or cure?. *Exp. Physiol.* 102 1335–1355. 10.1113/EP086283 28833689

[B48] Tournis S. Makris K. Cavalier E. Trovas G. (2020). Cardiovascular Risk in Patients with Primary Hyperparathyroidism. *Curr. Pharm. Des.* 26 5628–5636. 10.2174/1381612824999201105165642 33155899

[B49] Townsend R. Elliott-Sale K. J. Pinto A. J. Thomas C. Scott J. P. R. Currell K. (2016). Parathyroid Hormone Secretion Is Controlled by Both Ionized Calcium and Phosphate During Exercise and Recovery in Men. *J. Clin. Endocrinol. Metab.* 101 3231–3239. 10.1210/jc.2016-1848 27294328

[B50] Vainionpaa A. Korpelainen R. Vaananen H. K. Haapalahti J. Jamsa T. Leppaluoto J. (2009). Effect of impact exercise on bone metabolism. *Osteoporos. Int.* 20 1725–1733. 10.1007/s00198-009-0881-6 19262975

[B51] Valtonen R. I. P. Kiviniemi A. Hintsala H. E. Ryti N. R. I. Kenttä T. Huikuri H. V. (2018). Cardiovascular responses to cold and submaximal exercise in patients with coronary artery disease. *Am. J. Physiol. Regul. Integr. Comp. Physiol.* 315 R768–R776. 10.1152/ajpregu.00069.2018 29975565

[B52] Vasikaran S. D. Chubb S. A. (2016). The use of biochemical markers of bone turnover in the clinical management of primary and secondary osteoporosis. *Endocrine* 52 222–225. 10.1007/s12020-016-0900-2 26906711

[B53] Vlachopoulos D. Barker A. R. Ubago-Guisado E. Ortega F. B. Krustrup P. Metcalf B. (2018). The effect of 12-month participation in osteogenic and non-osteogenic sports on bone development in adolescent male athletes. The PRO-BONE study. *J. Sci. Med. Sport* 21 404–409. 10.1016/j.jsams.2017.08.018 28886923

[B54] Vlachopoulos D. Barker A. R. Williams C. A. ArngríMsson S. A. Knapp K. M. Metcalf B. S. (2017). The Impact of Sport Participation on Bone Mass and Geometry in Male Adolescents. *Med. Sci. Sports Exerc.* 49 317–326. 10.1249/mss.0000000000001091 27631395

[B55] Wein M. N. Kronenberg H. M. (2018). Regulation of Bone Remodeling by Parathyroid Hormone. *Cold Spring Harb. Perspect. Med.* 8:a031237. 10.1101/cshperspect.a031237 29358318 PMC6071549

