# Supplementary material for: Response of Bone Metabolism Markers to Ice Swimming in Regular Practitioners
Source: Front Physiol. 2021 Nov 26;12:731523. doi: 10.3389/fphys.2021.731523 (PMC8662563; doi:10.3389/fphys.2021.731523)
Supplement: Supplementary file 1 [file Data_Sheet_1.docx]

**Supplemental Table 1** Variations of bone metabolism characteristics responses to ice swimming according to age stratified.

| Serum Characteristic | N | Pre-IS | Post-IS | *P*-value^a^ | Change (Δ) |
| --- | --- | --- | --- | --- | --- |
| Age, 40-55 year, PTH, median (Q1-Q3), pg/mL | 32 | **38.140 (31.160-44.800)** | **56.285 (39.642-67.843)** | **<0.001** | **12.750 (7.473-22.587)** |
| Ca^2+^, median (Q1-Q3), mmol/L | 32 | **2.350 (2.308-2.382)** | **2.410 (2.330-2.470)** | **0.001** | **0.055 (0.005-0.112)** |
| Pi, mean (SD), mmol/L | 32 | **1.132 (0.190)** | **1.391 (0.191)** | **<0.001** | **0.260 (0.135)** |
| Mg^2+^, mean (SD), mmol/L | 32 | 1.011 (0.055) | 1.010 (0.067) | 0.932 | -0.001 (0.041) |
| N-MID, median (Q1-Q3), ng/mL | 32 | 14.275 (12.000-17.682) | 14.480 (11.705-18.465) | 0.169 | 0.265 (-0.328-1.020) |
| TPINP, median (Q1-Q3), ng/mL | 32 | **38.195 (33.285-53.195)** | **37.970 (30.680-50.627)** | **0.008** | **-1.185 (-2.697-0.145)** |
| β-CTX, median (Q1-Q3), ng/mL | 32 | 0.186 (0.164-0.235) | 0.188 (0.152-0.309) | 0.313 | -0.008 (-0.032-0.016) |
| Age, 56-65 year, PTH, median (Q1-Q3), pg/mL | 40 | **39.095 (30.935-47.602)** | **46.905 (37.913-61.070)** | **<0.001** | **8.285 (2.150-16.902)** |
| Ca2+, median (Q1-Q3), mmol/L | 40 | **2.340 (2.280-2.382)** | **2.410 (2.337-2.473)** | **<0.001** | **0.060 (0.025-0.110)** |
| Pi, mean (SD), mmol/L | 40 | **1.081 (0.143)** | **1.301 (0.154)** | **<0.001** | **0.220 (0.170)** |
| Mg2+, mean (SD), mmol/L | 40 | **1.000 (0.061)** | **1.016 (0.071)** | **0.017** | **0.016 (0.041)** |
| N-MID, median (Q1-Q3), ng/mL | 40 | 13.920 (11.885-17.695) | 14.390 (11.640-17.785) | 0.510 | -0.140 (-0.610-0.218) |
| TPINP, median (Q1-Q3), ng/mL | 40 | **40.245 (32.707-50.183)** | **38.905 (29.522-50.877)** | **<0.001** | **-2.490 (-4.170--0.222)** |
| β-CTX, median (Q1-Q3), ng/mL | 40 | **0.182 (0.147-0.278)** | **0.175 (0.133-0.273)** | **0.008** | **-0.011 (-0.028-0.003)** |
| Age, ≥66 year, PTH, median (Q1-Q3), pg/mL | 15 | 49.830 (42.270-55.410) | 52.250 (45.700-65.960) | 0.156 | 10.400 (-0.770-23.860) |
| Ca^2+^, mean (SD), mmol/L | 15 | **2.317 (0.095)** | **2.365 (0.080)** | **0.032** | **0.048 (0.078)** |
| Pi, mean (SD), mmol/L | 15 | **1.099 (0.152)** | **1.330 (0.198)** | **<0.001** | **0.231 (0.161)** |
| Mg^2+^, mean (SD), mmol/L | 15 | 1.019 (0.069) | 1.027 (0.067) | 0.471 | 0.009 (0.045) |
| N-MID, mean (SD), ng/mL | 15 | 12.325 (3.009) | 12.018 (2.859) | 0.181 | -0.307 (0.845) |
| TPINP, mean (SD), ng/mL | 15 | **34.381 (9.919)** | **32.140 (9.422)** | **0.022** | **-2.241 (3.367)** |
| β-CTX, median (Q1-Q3), ng/mL | 15 | **0.177 (0.146-0.213)** | **0.156 (0.145-0.172)** | **0.016** | **-0.020 (-0.032--0.002)** |

Abbreviations: SD: standard deviation; Q1, first quartile; Q3, third quartile; PTH, parathyroid hormone; N-MID, N-terminal osteocalcin; TPINP, total propeptide of procollagen 1; β-CTX, C-terminal crosslaps. ^a^The *P*-values were obtained using the paired samples t test or the Wilcoxon signed-rank tests to evaluate differences in serum bone metabolism markers before and after IS according to the type of variable.

**Supplemental Table 2** Variations of bone metabolism characteristics responses to ice swimming according to sex stratified.

| Serum Characteristic | N | Pre-IS | Post-IS | *P*-value | Change (Δ) |
| --- | --- | --- | --- | --- | --- |
| Male PTH, median (Q1-Q3), pg/mL | 72 | **39.865 (32.142-47.895)** | **47.920 (40.517-65.775)** | **<0.001** | **10.025 (2.405-18.407)** |
| Ca^2+^, median (Q1-Q3), mmol/L | 72 | **2.330 (2.277-2.380)** | **2.410 (2.330-2.462)** | **<0.001** | **0.060 (0.010-0.110)** |
| Pi, median (Q1-Q3), mmol/L | 72 | **1.100 (0.990-1.190)** | **1.340 (1.197-1.510)** | **<0.001** | **0.290 (0.147-0.360)** |
| Mg^2+^, mean (SD), mmol/L | 72 | **1.007 (0.061)** | **1.017 (0.069)** | **0.045** | **0.010 (0.043)** |
| N-MID, median (Q1-Q3), ng/mL | 72 | 13.585 (11.138-16.825) | 13.435 (11.405-17.082) | 0.902 | 0.030 (-0.480-0.463) |
| TPINP, median (Q1-Q3), ng/mL | 72 | **37.245 (31.218-44.445)** | **35.855 (29.190-41.288)** | **<0.001** | **-1.530 (-3.195-0.002)** |
| β-CTX, median (Q1-Q3), ng/mL | 72 | **0.182 (0.147-0.228)** | **0.168 (0.134-0.222)** | **<0.001** | **-0.012 (-0.031-0.002)** |
| Female PTH, median (Q1-Q3), pg/mL | 15 | **42.100 (36.005-52.090)** | **64.880 (47.980-70.680)** | **0.003** | **16.260 (9.780-21.545)** |
| Ca^2+^, median (Q1-Q3), mmol/L | 15 | **2.350 (2.310-2.385)** | **2.370 (2.335-2.475)** | **0.021** | **0.040 (-0.010-0.105)** |
| Pi, mean (SD), mmol/L | 15 | **1.127 (0.136)** | **1.297 (0.107)** | **<0.001** | **0.169 (0.144)** |
| Mg^2+^, mean (SD), mmol/L | 15 | 1.009 (0.056) | 1.009 (0.067) | 1.000 | 0.000 (0.034) |
| N-MID, median (Q1-Q3), ng/mL | 15 | 16.630 (12.230-19.685) | 16.500 (11.740-19.760) | 0.932 | -0.040 (-0.620-0.490) |
| TPINP, mean (SD), ng/mL | 15 | **65.337 (33.636)** | **59.288 (30.122)** | **0.002** | **-6.049 (6.285)** |
| β-CTX, median (Q1-Q3), ng/mL | 15 | 0.264 (0.168-0.384) | 0.246 (0.160-0.355) | 0.842 | -0.006 (-0.031-0.031) |

Abbreviations: SD: standard deviation; Q1, first quartile; Q3, third quartile; PTH, parathyroid hormone; N-MID, N-terminal osteocalcin; TPINP, total propeptide of procollagen 1; β-CTX, C-terminal crosslaps. ^a^The *P*-values were obtained using the paired samples t test or the Wilcoxon signed-rank tests to evaluate differences in serum bone metabolism markers before and after IS according to the type of variable.

**Supplemental Table 3.** The differences in bone metabolism characteristics among the normal bone mass, osteopenia, and osteoporosis groups in age 40-55 year participants.

| Characteristic | Normal (N =15) | Osteopenia (N =15) | Osteoporosis (N =2) | *P*-value^a^ | *P* for trend |
| --- | --- | --- | --- | --- | --- |
| Pre-IS PTH, median (Q1-Q3), pg/mL | 38.52 (30.95-43.85) | 39.37 (33.41-45.27) | 29.07 (25.28-32.86) | 0.412 | 0.429 |
| Pre-IS Ca^2+^, mean (SD), mmol/L | 2.35 (0.08) | 2.36 (0.08) | 2.33 (0.05) | 0.865 | 0.953 |
| Pre-IS Pi, mean (SD), mmol/L | 1.14 (0.13) | 1.12 (0.25) | 1.15 (0.10) | 0.975 | 0.953 |
| Pre-IS Mg^2+^, mean (SD), mmol/L | 1.02 (0.06) | 1.01 (0.05) | 0.98 (0.03) | 0.623 | 0.353 |
| Pre-IS N-MID, median (Q1-Q3), ng/mL | 12.80 (11.41-14.33) | 17.05 (13.93-17.87) | 16.09 (13.32-18.87) | 0.145 | 0.104 |
| Pre-IS TPINP, median (Q1-Q3), ng/mL | **34.07 (28.16-39.57)** | **45.63 (37.50-61.07)** | **54.62 (41.82-67.43)** | **0.028** | **0.018** |
| Pre-IS β-CTX, median (Q1-Q3), ng/mL | 0.18 (0.17-0.22) | 0.21 (0.14-0.31) | 0.27 (0.19-0.35) | 0.998 | 0.413 |
| ΔPTH, median (Q1-Q3), pg/mL | 16.87 (10.89-25.43) | 9.97 (4.92-17.44) | 24.80 (21.50-28.11) | 0.179 | 0.434 |
| ΔCa^2+^, mean (SD), mmol/L | 0.05 (0.07) | 0.06 (0.09) | 0.08 (0.16) | 0.971 | 0.720 |
| ΔPi, mean (SD), mmol/L | 0.25 (0.15) | 0.26 (0.13) | 0.35 (0.13) | 0.611 | 0.425 |
| ΔMg^2+^, mean (SD), mmol/L | -0.00 (0.03) | -0.00 (0.05) | 0.04 (0.01) | 0.464 | 0.479 |
| ΔN-MID, mean (SD), ng/mL | 0.10 (1.03) | 0.38 (1.24) | 0.78 (0.83) | 0.650 | 0.352 |
| ΔTPINP, mean (SD), ng/mL | -1.35 (2.91) | -1.38 (4.68) | 0.19 (0.90) | 0.857 | 0.746 |
| Δβ-CTX, mean (SD), ng/mL | -0.00 (0.03) | -0.01 (0.05) | 0.03 (0.05) | 0.468 | 0.868 |

Abbreviations: SD: standard deviation; Q1, first quartile; Q3, third quartile; Pre-IS, the circulating levels before ice swimming; PTH, parathyroid hormone; N-MID, N-terminal osteocalcin; TPINP, total propeptide of procollagen 1; β-CTX, C-terminal crosslaps; Δ, the circulating changes responses to ice swimming. ^a^The P-values were obtained using One-way ANOVA, Welch one-way ANOVA, or Kruskal-Wallis one-way ANOVA was used to evaluate differences among the normal bone mass, osteopenia, and osteoporosis groups according to the equality of error variances (Levene’s test) and the type of variable.

**Supplemental Table 4.** The differences in bone metabolism characteristics among the normal bone mass, osteopenia, and osteoporosis groups in age 56-65 year participants.

| Characteristic | Normal (N =17) | Osteopenia (N =15) | Osteoporosis (N =8) | *P*-value^a^ | *P* for trend |
| --- | --- | --- | --- | --- | --- |
| Pre-IS PTH, median (Q1-Q3), pg/mL | 40.12 (34.59-48.12) | 38.00 (30.41-44.31) | 38.29 (30.25-71.12) | 0.759 | 0.132 |
| Pre-IS Ca^2+^, median (Q1-Q3), mmol/L | **2.31 (2.28-2.35)** | **2.34 (2.29-2.38)** | **2.43 (2.35-2.51)** | **0.064** | **0.010** |
| Pre-IS Pi, mean (SD), mmol/L | 1.09 (0.11) | 1.09 (0.17) | 1.04 (0.18) | 0.641 | 0.403 |
| Pre-IS Mg^2+^, mean (SD), mmol/L | 0.98 (0.06) | 1.01 (0.06) | 1.02 (0.07) | 0.363 | 0.172 |
| Pre-IS N-MID, median (Q1-Q3), ng/mL | **12.55 (11.91-14.28)** | **16.45 (11.80-17.52)** | **17.92 (14.25-21.94)** | **0.180** | **0.032** |
| Pre-IS TPINP, mean (SD), ng/mL | **37.17 (12.06)** | **41.14 (9.90)** | **67.85 (41.45)** | **0.136** | **0.004** |
| Pre-IS β-CTX, mean (SD), ng/mL | 0.22 (0.08) | 0.19 (0.08) | 0.33 (0.23) | 0.227 | 0.133 |
| ΔPTH, median (Q1-Q3), pg/mL | 4.60 (-2.93-13.03) | 8.58 (6.59-16.70) | 12.68 (4.08-21.83) | 0.316 | 0.100 |
| ΔCa^2+^, median (Q1-Q3), mmol/L | 0.06 (0.03-0.11) | 0.05 (0.00-0.08) | 0.08 (0.05-0.11) | 0.271 | 0.414 |
| ΔPi, mean (SD), mmol/L | 0.17 (0.17) | 0.24 (0.17) | 0.29 (0.17) | 0.224 | 0.082 |
| ΔMg^2+^, mean (SD), mmol/L | 0.03 (0.04) | 0.00 (0.04) | 0.01 (0.03) | 0.137 | 0.194 |
| ΔN-MID, median (Q1-Q3), ng/mL | -0.19 (-0.72-0.10) | 0.05 (-0.45-0.38) | -0.22 (-0.53-0.54) | 0.646 | 0.497 |
| ΔTPINP, mean (SD), ng/mL | **-2.47 (2.81)** | **-0.66 (3.69)** | **-8.26 (8.09)** | **0.058** | **0.044** |
| Δβ-CTX, mean (SD), ng/mL | -0.01 (0.02) | -0.01 (0.03) | -0.01 (0.05) | 0.965 | 0.791 |

Abbreviations: SD: standard deviation; Q1, first quartile; Q3, third quartile; Pre-IS, the circulating levels before ice swimming; PTH, parathyroid hormone; N-MID, N-terminal osteocalcin; TPINP, total propeptide of procollagen 1; β-CTX, C-terminal crosslaps; Δ, the circulating changes responses to ice swimming. ^a^The P-values were obtained using One-way ANOVA, Welch one-way ANOVA, or Kruskal-Wallis one-way ANOVA was used to evaluate differences among the normal bone mass, osteopenia, and osteoporosis groups according to the equality of error variances (Levene’s test) and the type of variable.

**Supplemental Table 5.** The differences in bone metabolism characteristics among the osteopenia and osteoporosis groups in age ≥ 66 year participants.

| Characteristic | Osteopenia (N =5) | Osteoporosis (N =10) | *P*-value^a^ |
| --- | --- | --- | --- |
| Pre-IS PTH, median (Q1-Q3), pg/mL | **41.33 (37.82-43.21)** | **54.53 (46.25-58.45)** | **0.037** |
| Pre-IS Ca^2+^, mean (SD), mmol/L | 2.31 (0.04) | 2.32 (0.11) | 0.812 |
| Pre-IS Pi, mean (SD), mmol/L | 1.05 (0.15) | 1.12 (0.16) | 0.435 |
| Pre-IS Mg^2+^, mean (SD), mmol/L | 1.03 (0.07) | 1.01 (0.07) | 0.782 |
| Pre-IS N-MID, mean (SD), ng/mL | 12.38 (3.33) | 12.30 (3.03) | 0.962 |
| Pre-IS TPINP, median (Q1-Q3), ng/mL | 39.98 (29.40-43.57) | 35.19 (33.07-36.91) | 0.391 |
| Pre-IS β-CTX, mean (SD), ng/mL | 0.15 (0.03) | 0.20 (0.06) | 0.118 |
| ΔPTH, median (Q1-Q3), pg/mL | 11.16 (10.40-13.18) | 1.18 (-9.71-28.70) | 0.270 |
| ΔCa^2+^, median (Q1-Q3), mmol/L | 0.02 (-0.04-0.13) | 0.07 (0.04-0.10) | 0.902 |
| ΔPi, mean (SD), mmol/L | 0.24 (0.07) | 0.23 (0.19) | 0.906 |
| ΔMg^2+^, mean (SD), mmol/L | 0.01 (0.05) | 0.01 (0.04) | 0.760 |
| ΔN-MID, median (Q1-Q3), ng/mL | 0.06 (-0.43-0.25) | -0.07 (-0.70-0.14) | 0.624 |
| ΔTPINP, mean (SD), ng/mL | -4.21 (3.41) | -1.26 (3.04) | 0.112 |
| Δβ-CTX, mean (SD), ng/mL | -0.01 (0.01) | -0.02 (0.03) | 0.498 |

Abbreviations: SD: standard deviation; Q1, first quartile; Q3, third quartile; Pre-IS, the circulating levels before ice swimming; PTH, parathyroid hormone; N-MID, N-terminal osteocalcin; TPINP, total propeptide of procollagen 1; β-CTX, C-terminal crosslaps; Δ, the circulating changes responses to ice swimming. ^a^The P-values were obtained using the t test or the Kruskal-Wallis test was used to evaluate differences among the osteopenia and osteoporosis groups according to the equality of error variances (Levene’s test) and the type of variable.

**Supplemental Table 6.** The differences in bone metabolism characteristics among the normal bone mass, osteopenia, and osteoporosis groups in male.

| Characteristic | Normal (N =33) | Osteopenia (N =34) | Osteoporosis (N =5) | *P*-value^a^ | *P* for trend |
| --- | --- | --- | --- | --- | --- |
| Pre-IS PTH, median (Q1-Q3), pg/mL | 39.61 (34.36-45.16) | 43.45 (32.91-52.12) | 33.59 (27.86-36.65) | 0.188 | 0.772 |
| Pre-IS Ca^2+^, median (Q1-Q3), mmol/L | 2.32 (2.28-2.35) | 2.33 (2.28-2.39) | 2.38 (2.36-2.47) | 0.232 | 0.188 |
| Pre-IS Pi, mean (SD), mmol/L | 1.10 (0.12) | 1.11 (0.21) | 1.01 (0.16) | 0.481 | 0.644 |
| Pre-IS Mg^2+^, mean (SD), mmol/L | 1.01 (0.07) | 1.01 (0.06) | 1.00 (0.04) | 0.979 | 0.934 |
| Pre-IS N-MID, median (Q1-Q3), ng/mL | 12.54 (10.92-15.35) | 14.59 (11.73-17.50) | 14.99 (12.02-16.31) | 0.347 | 0.368 |
| Pre-IS TPINP, median (Q1-Q3), ng/mL | 34.60 (29.29-40.59) | 37.39 (33.95-45.33) | 43.29 (38.19-47.82) | 0.258 | 0.081 |
| Pre-IS β-CTX, median (Q1-Q3), ng/mL | 0.18 (0.15-0.22) | 0.19 (0.14-0.24) | 0.16 (0.14-0.18) | 0.808 | 0.827 |
| ΔPTH, median (Q1-Q3), pg/mL | 11.27 (2.36-20.45) | 7.49 (1.81-15.59) | 16.72 (8.63-27.10) | 0.341 | 0.554 |
| ΔCa^2+^, mean (SD), mmol/L | 0.06 (0.07) | 0.06 (0.07) | 0.06 (0.06) | 0.941 | 0.774 |
| ΔPi, mean (SD), mmol/L | 0.23 (0.15) | 0.25 (0.16) | 0.39 (0.10) | 0.088 | 0.066 |
| ΔMg^2+^, mean (SD), mmol/L | 0.02 (0.04) | 0.00 (0.05) | 0.01 (0.02) | 0.405 | 0.316 |
| ΔN-MID, median (Q1-Q3), ng/mL | 0.02 (-0.47-0.44) | 0.04 (-0.53-0.47) | -0.10 (-0.33-0.45) | 0.964 | 0.840 |
| ΔTPINP, median (Q1-Q3), ng/mL | -1.80 (-3.19--0.47) | -0.97 (-3.11-0.33) | -0.44 (-2.09-0.46) | 0.544 | 0.344 |
| Δβ-CTX, median (Q1-Q3), ng/mL | -0.01 (-0.02-0.00) | -0.03 (-0.04-0.00) | -0.01 (-0.01--0.01) | 0.158 | 0.717 |

Abbreviations: SD: standard deviation; Q1, first quartile; Q3, third quartile; Pre-IS, the circulating levels before ice swimming; PTH, parathyroid hormone; N-MID, N-terminal osteocalcin; TPINP, total propeptide of procollagen 1; β-CTX, C-terminal crosslaps; Δ, the circulating changes responses to ice swimming. ^a^The P-values were obtained using One-way ANOVA, Welch one-way ANOVA, or Kruskal-Wallis one-way ANOVA was used to evaluate differences among the normal bone mass, osteopenia, and osteoporosis groups according to the equality of error variances (Levene’s test) and the type of variable.

**Supplemental Table 7.** The differences in bone metabolism characteristics among the normal bone mass, osteopenia, and osteoporosis groups in female.

| Characteristic | Normal (N =4) | Osteopenia (N =6) | Osteoporosis (N =5) | *P*-value^a^ | P for trend |
| --- | --- | --- | --- | --- | --- |
| Pre-IS PTH, mean (SD), pg/mL | 44.72 (9.64) | 41.75 (8.00) | 81.29 (67.67) | 0.472 | 0.172 |
| Pre-IS Ca^2+^, median (Q1-Q3), mmol/L | 2.35 (2.32-2.36) | 2.33 (2.33-2.37) | 2.39 (2.29-2.58) | 0.617 | 0.142 |
| Pre-IS Pi, median (Q1-Q3), mmol/L | 1.15 (1.14-1.19) | 1.06 (1.02-1.12) | 1.08 (1.08-1.17) | 0.362 | 0.455 |
| Pre-IS Mg^2+^, mean (SD), mmol/L | 0.99 (0.05) | 1.02 (0.04) | 1.01 (0.08) | 0.702 | 0.525 |
| Pre-IS N-MID, median (Q1-Q3), ng/mL | 12.93 (12.39-13.66) | 17.08 (13.02-18.12) | 19.84 (19.53-28.23) | 0.256 | 0.092 |
| Pre-IS TPINP, mean (SD), ng/mL | **41.45 (14.34)** | **64.85 (25.98)** | **85.03 (43.66)** | **0.156** | **0.049** |
| Pre-IS β-CTX, mean (SD), ng/mL | 0.26 (0.11) | 0.21 (0.10) | 0.43 (0.24) | 0.103 | 0.134 |
| ΔPTH, mean (SD), pg/mL | 14.38 (14.47) | 18.64 (8.82) | 22.80 (39.98) | 0.881 | 0.607 |
| ΔCa^2+^, median (Q1-Q3), mmol/L | **0.00 (-0.03-0.05)** | **0.00 (-0.01-0.04)** | **0.11 (0.06-0.20)** | **0.058** | **0.033** |
| ΔPi, mean (SD), mmol/L | 0.08 (0.11) | 0.18 (0.13) | 0.22 (0.17) | 0.364 | 0.165 |
| ΔMg^2+^, mean (SD), mmol/L | -0.01 (0.03) | -0.01 (0.03) | 0.02 (0.03) | 0.149 | 0.116 |
| ΔN-MID, mean (SD), ng/mL | -0.92 (0.95) | 0.49 (1.24) | 0.50 (1.40) | 0.187 | 0.126 |
| ΔTPINP, median (Q1-Q3), ng/mL | -2.98 (-4.47--2.71) | -3.25 (-5.22--0.71) | -12.59 (-13.63--8.89) | 0.144 | 0.073 |
| Δβ-CTX, mean (SD), ng/mL | -0.01 (0.03) | 0.00 (0.03) | -0.01 (0.06) | 0.821 | 0.875 |

Abbreviations: SD: standard deviation; Q1, first quartile; Q3, third quartile; Pre-IS, the circulating levels before ice swimming; PTH, parathyroid hormone; N-MID, N-terminal osteocalcin; TPINP, total propeptide of procollagen 1; β-CTX, C-terminal crosslaps; Δ, the circulating changes responses to ice swimming. ^a^The P-values were obtained using One-way ANOVA, Welch one-way ANOVA, or Kruskal-Wallis one-way ANOVA was used to evaluate differences among the normal bone mass, osteopenia, and osteoporosis groups according to the equality of error variances (Levene’s test) and the type of variable.

**Supplemental Table 8.** Correlations between bone metabolism characteristics and bone mineral parameters and potential fracture risk in all subjects.

| Characteristic | L1-L4 BMC | L1-L4 BMD | FN BMC | FN BMD | FRAX HF1 | FRAX HF2 | FRAX MOF1 | FRAX MOF2 |
| --- | --- | --- | --- | --- | --- | --- | --- | --- |
| Pre-IS PTH, (pg/mL) | -0.0250 | 0.0290 | -0.0646 | 0.0102 | 0.1890 | 0.0616 | 0.1741 | 0.0507 |
| Pre-IS Ca^2+^, (mmol/L) | -0.1867 | -0.1922 | -0.1029 | -0.1609 | -0.0376 | 0.0995 | -0.0279 | 0.0828 |
| Pre-IS Pi, (mmol/L) | 0.0297 | 0.0583 | -0.0207 | -0.0183 | -0.1157 | -0.0128 | -0.0124 | 0.0517 |
| Pre-IS Mg^2+^, (mmol/L) | -0.0383 | -0.0873 | 0.0516 | 0.0610 | 0.0712 | 0.0041 | 0.1202 | -0.0202 |
| Pre-IS N-MID, (ng/mL) | **-0.2566*** | **-0.2832**** | -0.0725 | -0.1010 | -0.0997 | 0.0210 | -0.1008 | -0.0528 |
| Pre-IS TPINP, (ng/mL) | **-0.3073**** | **-0.2639*** | -0.1952 | -0.1667 | -0.1414 | 0.0118 | -0.1672 | -0.0826 |
| Pre-IS β-CTX, (ng/mL) | -0.1971 | -0.1134 | -0.0795 | 0.0328 | -0.0985 | -0.1086 | -0.0725 | -0.1023 |
| ΔPTH, (pg/mL) | -0.1258 | -0.1165 | 0.0160 | 0.0880 | -0.0815 | -0.1122 | -0.0360 | -0.0894 |
| ΔCa^2+^, (mmol/L) | -0.0351 | -0.0886 | -0.0479 | -0.0324 | 0.1170 | 0.0812 | 0.1595 | 0.0964 |
| ΔPi, (mmol/L) | -0.1122 | -0.1646 | -0.0670 | -0.0792 | -0.0178 | 0.1153 | -0.0931 | 0.0013 |
| ΔMg^2+^, (mmol/L) | 0.0055 | 0.0354 | -0.1109 | -0.0765 | 0.1416 | 0.1343 | 0.1627 | 0.1264 |
| ΔN-MID, (ng/mL) | -0.0487 | -0.0819 | -0.0449 | -0.0642 | -0.1233 | -0.0035 | -0.0957 | -0.0447 |
| ΔTPINP, (ng/mL) | 0.1017 | 0.0740 | 0.1746 | 0.0815 | -0.0964 | -0.0779 | -0.0758 | -0.0630 |
| Δβ-CTX, (ng/mL) | 0.0971 | 0.0784 | 0.1045 | 0.0883 | -0.1289 | -0.0942 | -0.1000 | -0.0464 |

Abbreviations: L1-L4, lumbar spine1-4; FN, femoral neck; BMC, bone mineral content; BMD, bone mineral density; FRAX HF1, FRAX predicted probability of hip fracture (without BMD); FRAX HF2, FRAX predicted probability of hip fracture (with BMD); FRAX MOF1, FRAX predicted probability of major osteoporotic fracture (without BMD); FRAX MOF2, FRAX predicted probability of major osteoporotic fracture (with BMD); Pre-IS, the circulating levels before ice swimming; PTH, parathyroid hormone; N-MID, N-terminal osteocalcin; TPINP, total propeptide of procollagen 1; β-CTX, C-terminal crosslaps; Δ, the circulating changes responses to ice swimming; The *P*-values were obtained using Spearman correlation coefficient analysis to determine the correlations between bone metabolism characteristics and bone mineral parameters and potential fracture risk. * *P* < 0.05; ** *P* < 0.01.

**Supplemental Table 9.** Correlations between bone metabolism characteristics and bone mineral parameters and potential fracture risk in age 40-55 year participants.

| Characteristic | L1-L4 BMC | L1-L4 BMD | FN BMC | FN BMD | FRAX HF1 | FRAX HF2 | FRAX MOF1 | FRAX MOF2 |
| --- | --- | --- | --- | --- | --- | --- | --- | --- |
| Pre-IS PTH, (pg/mL) | 0.0357 | 0.0462 | 0.2834 | 0.3083 | -0.1310 | -0.2985 | -0.0487 | -0.1702 |
| Pre-IS Ca^2+^, (mmol/L) | 0.0396 | 0.1256 | -0.0885 | -0.1785 | -0.0550 | 0.0709 | -0.0182 | 0.0338 |
| Pre-IS Pi, (mmol/L) | -0.0351 | 0.1460 | 0.0050 | -0.0011 | -0.0063 | -0.0374 | 0.0322 | 0.1594 |
| Pre-IS Mg^2+^, (mmol/L) | 0.0799 | 0.0980 | 0.2163 | 0.2942 | 0.0540 | -0.2372 | 0.3188 | 0.1258 |
| Pre-IS N-MID, (ng/mL) | -0.3028 | -0.3112 | -0.1487 | -0.1749 | -0.0556 | 0.1802 | 0.0262 | 0.0956 |
| Pre-IS TPINP, (ng/mL) | -0.3246 | -0.3013 | -0.2770 | -0.3372 | -0.1958 | 0.2253 | -0.2521 | -0.0515 |
| Pre-IS β-CTX, (ng/mL) | -0.1461 | -0.0735 | 0.0930 | 0.2471 | -0.2126 | -0.2528 | -0.1824 | -0.2250 |
| ΔPTH, (pg/mL) | -0.0084 | -0.0194 | 0.1683 | 0.2060 | -0.0776 | -0.2463 | 0.0207 | -0.0461 |
| ΔCa^2+^, (mmol/L) | 0.1229 | 0.0405 | 0.1461 | 0.1985 | 0.0138 | -0.1890 | 0.0264 | -0.1934 |
| ΔPi, (mmol/L) | 0.0083 | -0.0937 | -0.1285 | -0.0792 | -0.2296 | 0.0479 | -0.1874 | -0.0183 |
| ΔMg^2+^, (mmol/L) | -0.0392 | -0.0790 | -0.1161 | -0.0613 | 0.1478 | 0.1249 | 0.1098 | 0.1599 |
| ΔN-MID, (ng/mL) | -0.0563 | -0.1239 | -0.0711 | -0.1371 | 0.0934 | 0.1155 | 0.2349 | 0.3275 |
| ΔTPINP, (ng/mL) | 0.1353 | 0.0979 | 0.0959 | -0.0759 | **0.3786*** | 0.1297 | **0.4388*** | **0.4096*** |
| Δβ-CTX, (ng/mL) | 0.1850 | 0.1041 | 0.1798 | 0.0319 | 0.1012 | 0.0734 | 0.2013 | 0.2067 |

Abbreviations: L1-L4, lumbar spine1-4; FN, femoral neck; BMC, bone mineral content; BMD, bone mineral density; FRAX HF1, FRAX predicted probability of hip fracture (without BMD); FRAX HF2, FRAX predicted probability of hip fracture (with BMD); FRAX MOF1, FRAX predicted probability of major osteoporotic fracture (without BMD); FRAX MOF2, FRAX predicted probability of major osteoporotic fracture (with BMD); Pre-IS, the circulating levels before ice swimming; PTH, parathyroid hormone; N-MID, N-terminal osteocalcin; TPINP, total propeptide of procollagen 1; β-CTX, C-terminal crosslaps; Δ, the circulating changes responses to ice swimming; The *P*-values were obtained using Spearman correlation coefficient analysis to determine the correlations between bone metabolism characteristics and bone mineral parameters and potential fracture risk. * *P* < 0.05.

**Supplemental Table 10.** Correlations between bone metabolism characteristics and bone mineral parameters and potential fracture risk in age 56-65 year participants.

| Characteristic | L1-L4 BMC | L1-L4 BMD | FN BMC | FN BMD | FRAX HF1 | FRAX HF2 | FRAX MOF1 | FRAX MOF2 |
| --- | --- | --- | --- | --- | --- | --- | --- | --- |
| Pre-IS PTH, (pg/mL) | -0.0426 | 0.0442 | -0.1247 | -0.0016 | 0.1912 | -0.0148 | 0.2158 | 0.0840 |
| Pre-IS Ca^2+^, (mmol/L) | -0.2848 | **-0.3516*** | -0.2807 | **-0.3658*** | 0.1390 | **0.3439*** | 0.1396 | 0.2871 |
| Pre-IS Pi, (mmol/L) | 0.0914 | 0.0318 | -0.0089 | -0.0072 | -0.1311 | -0.0600 | -0.0535 | -0.1049 |
| Pre-IS Mg^2+^, (mmol/L) | -0.0878 | -0.1478 | -0.2220 | -0.2605 | 0.1125 | 0.2169 | -0.0023 | 0.0080 |
| Pre-IS N-MID, (ng/mL) | -0.2276 | -0.2615 | -0.1599 | -0.2056 | 0.1114 | 0.1593 | 0.0006 | -0.0054 |
| Pre-IS TPINP, (ng/mL) | **-0.3548*** | **-0.3342*** | **-0.3206*** | -0.1997 | 0.0399 | 0.0765 | 0.0641 | 0.0844 |
| Pre-IS β-CTX, (ng/mL) | -0.2596 | -0.1212 | -0.1870 | -0.0797 | 0.0647 | 0.0510 | 0.0902 | -0.0039 |
| ΔPTH, (pg/mL) | -0.2169 | -0.2018 | **-0.3504*** | -0.2408 | 0.1059 | 0.1546 | 0.1016 | 0.0935 |
| ΔCa^2+^, (mmol/L) | -0.2303 | -0.1839 | -0.0446 | -0.0801 | **0.3464*** | 0.1901 | **0.3767*** | 0.2783 |
| ΔPi, (mmol/L) | -0.1838 | -0.1534 | -0.1258 | -0.1552 | 0.1549 | 0.2682 | -0.0251 | 0.0865 |
| ΔMg^2+^, (mmol/L) | -0.0807 | 0.0301 | 0.0653 | 0.0796 | 0.0205 | -0.0247 | 0.0542 | -0.0367 |
| ΔN-MID, (ng/mL) | -0.1108 | -0.1116 | -0.0809 | -0.1292 | 0.0552 | 0.1290 | 0.0201 | -0.0218 |
| ΔTPINP, (ng/mL) | 0.2846 | 0.2766 | 0.2604 | 0.1817 | -0.1534 | -0.1594 | **-0.3419*** | -0.2938 |
| Δβ-CTX, (ng/mL) | 0.0772 | -0.0100 | 0.0638 | 0.1050 | -0.1961 | -0.1970 | -0.2004 | -0.1491 |

Abbreviations: L1-L4, lumbar spine1-4; FN, femoral neck; BMC, bone mineral content; BMD, bone mineral density; FRAX HF1, FRAX predicted probability of hip fracture (without BMD); FRAX HF2, FRAX predicted probability of hip fracture (with BMD); FRAX MOF1, FRAX predicted probability of major osteoporotic fracture (without BMD); FRAX MOF2, FRAX predicted probability of major osteoporotic fracture (with BMD); Pre-IS, the circulating levels before ice swimming; PTH, parathyroid hormone; N-MID, N-terminal osteocalcin; TPINP, total propeptide of procollagen 1; β-CTX, C-terminal crosslaps; Δ, the circulating changes responses to ice swimming; The *P*-values were obtained using Spearman correlation coefficient analysis to determine the correlations between bone metabolism characteristics and bone mineral parameters and potential fracture risk. * *P* < 0.05.

**Supplemental Table 11.** Correlations between bone metabolism characteristics and bone mineral parameters and potential fracture risk in age ≥66 year participants.

| Characteristic | L1-L4 BMC | L1-L4 BMD | FN BMC | FN BMD | FRAX HF1 | FRAX HF2 | FRAX MOF1 | FRAX MOF2 |
| --- | --- | --- | --- | --- | --- | --- | --- | --- |
| Pre-IS PTH, (pg/mL) | -0.3107 | -0.3321 | **-0.6893**** | **-0.6286*** | -0.1813 | 0.4534 | 0.0877 | 0.4347 |
| Pre-IS Ca^2+^, (mmol/L) | -0.1578 | -0.1722 | **0.5292*** | **0.5614*** | 0.4653 | -0.3411 | 0.1826 | -0.2004 |
| Pre-IS Pi, (mmol/L) | 0.2200 | 0.0465 | -0.1485 | -0.1664 | 0.0315 | 0.2917 | 0.1955 | 0.3324 |
| Pre-IS Mg^2+^, (mmol/L) | -0.0985 | -0.1719 | **0.5640*** | **0.5622*** | 0.4942 | -0.2174 | 0.1113 | -0.2861 |
| Pre-IS N-MID, (ng/mL) | -0.0357 | -0.1607 | 0.4571 | 0.3429 | 0.3878 | -0.0860 | 0.1307 | -0.0483 |
| Pre-IS TPINP, (ng/mL) | 0.4464 | 0.3357 | 0.2536 | 0.2286 | -0.0987 | -0.1308 | -0.2381 | -0.3309 |
| Pre-IS β-CTX, (ng/mL) | -0.0250 | -0.3464 | -0.2607 | -0.4214 | 0.0916 | 0.3602 | 0.0430 | 0.3417 |
| ΔPTH, (pg/mL) | 0.0429 | 0.0750 | **0.5893*** | **0.6464*** | 0.5027 | -0.3638 | 0.0895 | -0.3256 |
| ΔCa^2+^, (mmol/L) | 0.1415 | 0.0251 | -0.3474 | -0.3903 | 0.1449 | **0.5750*** | 0.1777 | 0.1776 |
| ΔPi, (mmol/L) | -0.2994 | -0.2200 | 0.3120 | 0.2687 | **0.6519**** | 0.0661 | 0.4195 | 0.1671 |
| ΔMg^2+^, (mmol/L) | 0.1498 | 0.2599 | **-0.5397*** | -0.4152 | -0.0145 | 0.5100 | 0.1376 | 0.1103 |
| ΔN-MID, (ng/mL) | 0.3536 | 0.1929 | 0.0536 | 0.1464 | -0.2208 | -0.2688 | -0.4799 | -0.4884 |
| ΔTPINP, (ng/mL) | -0.4536 | -0.5036 | 0.1643 | 0.1321 | 0.0664 | -0.1649 | 0.1343 | -0.0537 |
| Δβ-CTX, (ng/mL) | 0.2131 | 0.3187 | -0.0179 | 0.1665 | -0.2016 | -0.1716 | -0.2011 | -0.1740 |

Abbreviations: L1-L4, lumbar spine1-4; FN, femoral neck; BMC, bone mineral content; BMD, bone mineral density; FRAX HF1, FRAX predicted probability of hip fracture (without BMD); FRAX HF2, FRAX predicted probability of hip fracture (with BMD); FRAX MOF1, FRAX predicted probability of major osteoporotic fracture (without BMD); FRAX MOF2, FRAX predicted probability of major osteoporotic fracture (with BMD); Pre-IS, the circulating levels before ice swimming; PTH, parathyroid hormone; N-MID, N-terminal osteocalcin; TPINP, total propeptide of procollagen 1; β-CTX, C-terminal crosslaps; Δ, the circulating changes responses to ice swimming; The *P*-values were obtained using Spearman correlation coefficient analysis to determine the correlations between bone metabolism characteristics and bone mineral parameters and potential fracture risk. * *P* < 0.05; ** *P* < 0.01.

**Supplemental Table 12.** Correlations between bone metabolism characteristics and bone mineral parameters and potential fracture risk in male.

| Characteristic | L1-L4 BMC | L1-L4 BMD | FN BMC | FN BMD | FRAX HF1 | FRAX HF2 | FRAX MOF1 | FRAX MOF2 |
| --- | --- | --- | --- | --- | --- | --- | --- | --- |
| Pre-IS PTH, (pg/mL) | 0.0590 | 0.1047 | 0.0077 | 0.0668 | 0.0909 | 0.0014 | 0.0492 | -0.0764 |
| Pre-IS Ca^2+^, (mmol/L) | -0.1937 | -0.2002 | -0.0538 | -0.1265 | -0.0757 | 0.0651 | -0.0676 | 0.0779 |
| Pre-IS Pi, (mmol/L) | 0.0308 | 0.0361 | 0.0167 | -0.0043 | -0.1258 | -0.0091 | -0.0311 | 0.0405 |
| Pre-IS Mg^2+^, (mmol/L) | -0.0196 | -0.0619 | 0.1018 | 0.1122 | 0.0171 | -0.0500 | 0.0621 | -0.1008 |
| Pre-IS N-MID, (ng/mL) | -0.1476 | -0.1754 | 0.0997 | 0.0818 | -0.1634 | -0.1138 | -0.1996 | -0.1756 |
| Pre-IS TPINP, (ng/mL) | -0.1498 | -0.1391 | 0.0718 | 0.0440 | -0.2107 | -0.0989 | **-0.3114**** | **-0.2679*** |
| Pre-IS β-CTX, (ng/mL) | -0.1026 | -0.0524 | 0.0946 | 0.1815 | -0.2029 | -0.2057 | -0.2036 | **-0.2398*** |
| ΔPTH, (pg/mL) | -0.0557 | -0.0786 | 0.1516 | 0.1676 | -0.1302 | -0.1237 | -0.1215 | -0.1631 |
| ΔCa^2+^, (mmol/L) | -0.0334 | -0.0341 | -0.1101 | -0.0600 | 0.0629 | 0.0419 | 0.1013 | 0.0373 |
| ΔPi, (mmol/L) | **-0.2396*** | -0.2106 | **-0.2554*** | -0.1695 | 0.0091 | 0.1223 | -0.0177 | 0.1012 |
| ΔMg^2+^, (mmol/L) | -0.0332 | 0.0477 | -0.2158 | -0.1453 | 0.1141 | 0.1360 | 0.1593 | 0.1322 |
| ΔN-MID, (ng/mL) | -0.0444 | -0.0171 | -0.0220 | -0.0007 | -0.1341 | -0.0794 | -0.0849 | -0.0611 |
| ΔTPINP, (ng/mL) | -0.0877 | -0.0717 | 0.0008 | -0.0660 | -0.0210 | 0.0384 | 0.0798 | 0.1362 |
| Δβ-CTX, (ng/mL) | 0.1532 | 0.1403 | **0.2356*** | 0.2145 | -0.1088 | -0.1684 | -0.0869 | -0.0782 |

Abbreviations: L1-L4, lumbar spine1-4; FN, femoral neck; BMC, bone mineral content; BMD, bone mineral density; FRAX HF1, FRAX predicted probability of hip fracture (without BMD); FRAX HF2, FRAX predicted probability of hip fracture (with BMD); FRAX MOF1, FRAX predicted probability of major osteoporotic fracture (without BMD); FRAX MOF2, FRAX predicted probability of major osteoporotic fracture (with BMD); Pre-IS, the circulating levels before ice swimming; PTH, parathyroid hormone; N-MID, N-terminal osteocalcin; TPINP, total propeptide of procollagen 1; β-CTX, C-terminal crosslaps; Δ, the circulating changes responses to ice swimming; The *P*-values were obtained using Spearman correlation coefficient analysis to determine the correlations between bone metabolism characteristics and bone mineral parameters and potential fracture risk. * *P* < 0.05; ** *P* < 0.01; *** *P* < 0.001.

**Supplemental Table 13.** Correlations between bone metabolism characteristics and bone mineral parameters and potential fracture risk in female.

| Characteristic | L1-L4 BMC | L1-L4 BMD | FN BMC | FN BMD | FRAX HF1 | FRAX HF2 | FRAX MOF1 | FRAX MOF2 |
| --- | --- | --- | --- | --- | --- | --- | --- | --- |
| Pre-IS PTH, (pg/mL) | -0.1821 | -0.1571 | -0.1214 | -0.0357 | **0.7080**** | 0.2549 | **0.7256**** | **0.5500*** |
| Pre-IS Ca^2+^, (mmol/L) | -0.2165 | -0.2755 | -0.3685 | -0.2773 | 0.2169 | 0.1799 | 0.2211 | 0.1145 |
| Pre-IS Pi, (mmol/L) | 0.3832 | 0.3706 | 0.1629 | 0.0483 | 0.0189 | -0.0081 | -0.0063 | 0.0412 |
| Pre-IS Mg^2+^, (mmol/L) | -0.0180 | -0.1400 | -0.2424 | -0.1185 | 0.2864 | 0.2031 | 0.3720 | 0.3303 |
| Pre-IS N-MID, (ng/mL) | **-0.5250*** | -0.5179 | **-0.6214*** | **-0.6286*** | 0.2246 | **0.5242*** | 0.1912 | 0.2786 |
| Pre-IS TPINP, (ng/mL) | -0.4286 | **-0.5286*** | **-0.6429*** | **-0.5750*** | 0.0755 | 0.4381 | 0.0518 | 0.1679 |
| Pre-IS β-CTX, (ng/mL) | -0.4464 | -0.2393 | -0.4679 | -0.3071 | 0.3432 | 0.3142 | 0.3074 | 0.2929 |
| ΔPTH, (pg/mL) | -0.1179 | -0.0286 | 0.0393 | 0.0536 | 0.1725 | -0.0754 | 0.1340 | -0.1000 |
| ΔCa^2+^, (mmol/L) | **-0.5220*** | **-0.5722*** | -0.3570 | -0.2888 | 0.4468 | 0.3273 | 0.5036 | 0.4879 |
| ΔPi, (mmol/L) | -0.3324 | -0.4021 | -0.2931 | -0.2824 | -0.2437 | 0.1024 | -0.2388 | -0.1787 |
| ΔMg^2+^, (mmol/L) | -0.2365 | -0.2924 | -0.0451 | -0.0632 | 0.3497 | 0.1162 | 0.3641 | 0.3195 |
| ΔN-MID, (ng/mL) | -0.2107 | -0.4750 | -0.4500 | -0.4000 | -0.0593 | 0.2891 | 0.0536 | 0.1786 |
| ΔTPINP, (ng/mL) | 0.4036 | 0.4929 | 0.4714 | 0.5143 | -0.5032 | **-0.6266*** | -0.4397 | **-0.5464*** |
| Δβ-CTX, (ng/mL) | 0.2571 | -0.1071 | -0.0643 | -0.2000 | -0.0557 | 0.1688 | -0.0143 | 0.0679 |

Abbreviations: L1-L4, lumbar spine1-4; FN, femoral neck; BMC, bone mineral content; BMD, bone mineral density; FRAX HF1, FRAX predicted probability of hip fracture (without BMD); FRAX HF2, FRAX predicted probability of hip fracture (with BMD); FRAX MOF1, FRAX predicted probability of major osteoporotic fracture (without BMD); FRAX MOF2, FRAX predicted probability of major osteoporotic fracture (with BMD); Pre-IS, the circulating levels before ice swimming; PTH, parathyroid hormone; N-MID, N-terminal osteocalcin; TPINP, total propeptide of procollagen 1; β-CTX, C-terminal crosslaps; Δ, the circulating changes responses to ice swimming; The *P*-values were obtained using Spearman correlation coefficient analysis to determine the correlations between bone metabolism characteristics and bone mineral parameters and potential fracture risk. * *P* < 0.05; ** *P* < 0.01; *** *P* < 0.001.
